# Characterization of an unusual SARS-CoV-2 main protease natural variant exhibiting resistance to nirmatrelvir and ensitrelvir

**DOI:** 10.1038/s42003-025-08487-w

**Published:** 2025-07-17

**Authors:** Dipendra Bhandari, Oksana Gerlits, Stephen Keable, Leighton Coates, Annie Aniana, Rodolfo Ghirlando, Nashaat T. Nashed, Andrey Kovalevsky, John M. Louis

**Affiliations:** 1 Neutron Scattering Division, Oak Ridge National Laboratory, Oak Ridge, TN USA; 2 Department of Natural Sciences, Tennessee Wesleyan University, Athens, TN USA; 3 Second Target Station, Oak Ridge National Laboratory, Oak Ridge, TN USA; 4 Laboratory of Chemical Physics, National Institute of Diabetes and Digestive and Kidney Diseases, National Institutes of Health, DHHS, Bethesda, MD USA; 5 Laboratory of Molecular Biology, National Institute of Diabetes and Digestive and Kidney Diseases, National Institutes of Health, DHHS, Bethesda, MD USA

**Keywords:** Proteases, X-ray crystallography

## Abstract

We investigate the effects of two naturally selected substitution and deletion (Δ) mutations, constituting part of the substrate binding subsites S2 and S4, on the structure, function, and inhibition of SARS CoV-2 main protease. Comparable to wild-type, MPro^D48Y/ΔP168^ undergoes N-terminal autoprocessing essential for stable dimer formation and mature-like catalytic activity. The structures are similar, but for an open active site conformation in MPro^D48Y/ΔP168^ and increased dynamics of the S2 helix, S5 loop, and the helical domain. Some dimer interface contacts exhibit shorter H bond distances corroborating the ~40-fold enhanced dimerization of the mutant although its thermal sensitivity to unfolding is 8 °C lower, relative to wild-type. ITC reveals a 3- and 5-fold decrease in binding affinity for nirmatrelvir and ensitrelvir, respectively, and similar GC373 affinity, to MPro^D48Y/ΔP168^ relative to wild-type. Structural differences in four inhibitor complexes of MPro^D48Y/ΔP168^ compared to wild-type are described. Consistent with enhanced dynamics, the S2 helix and S5 loop adopting a more open conformation appears to be a unique feature of MPro^D48Y/ΔP168^ both in the inhibitor-free and bound states. Our results suggest that mutational effects are compensated by changes in the conformational dynamics and thereby modulate N-terminal autoprocessing, K_dimer_, catalytic efficiency, and inhibitor binding.

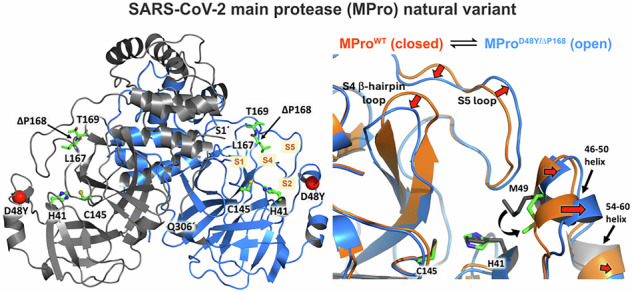

## Abstract (web summary)

Drug resistance mutations lead to active site opening and changes in conformational dynamics which modulate N-terminal autoprocessing and parameters that govern dimerization, catalytic function, and inhibition of SARS-CoV-2 main protease.

## Introduction

**Fig. 1:**
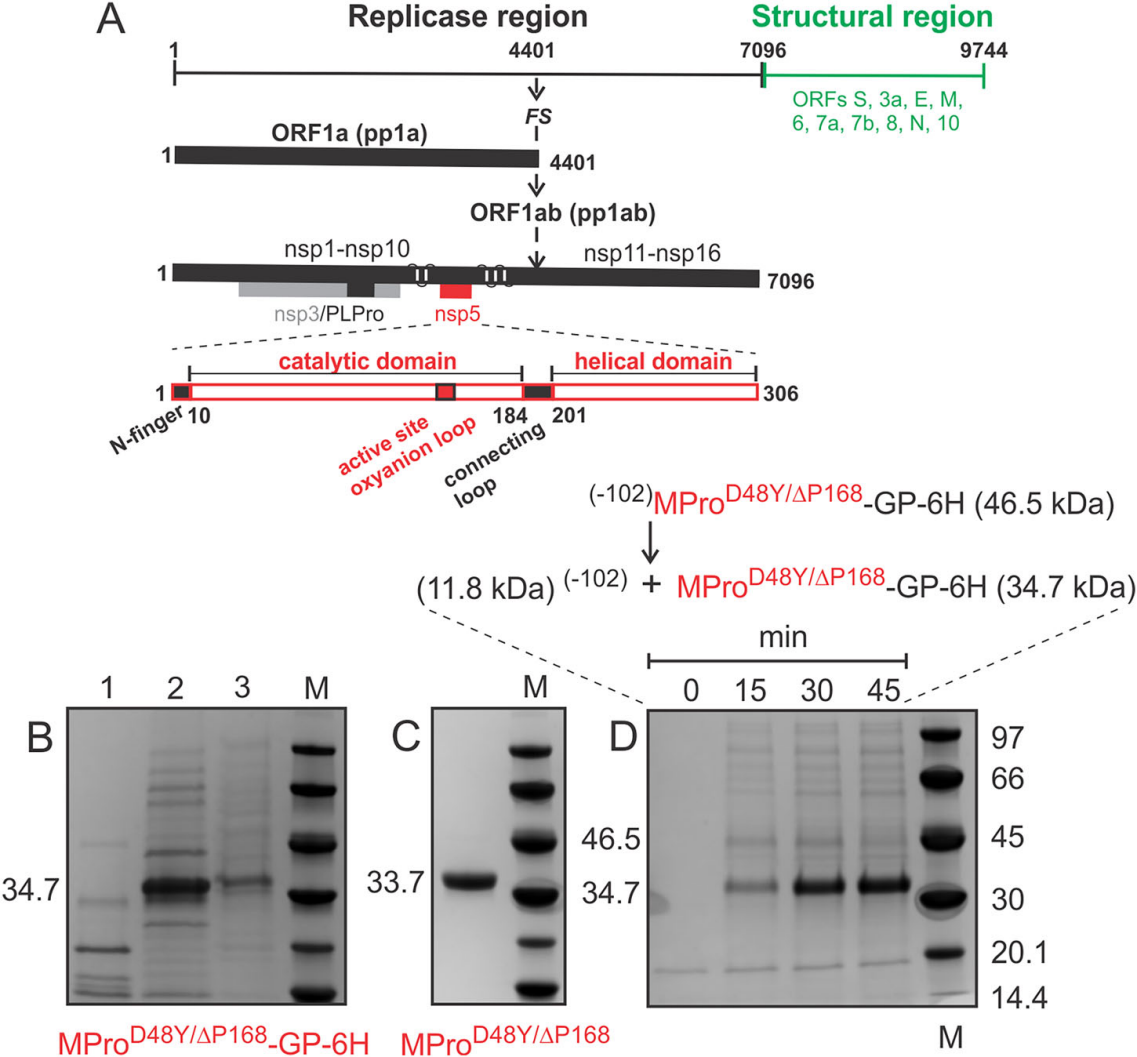
Genome organization of SARS-CoV-2, purification of MPro^D48Y/ΔP168^ dimer and autoprocessing of ^(−102)^MPro^D48Y/ΔP168^-GP-6H precursor. **A** The ~30 kb genome codes for the various proteins in at least 12 open reading frames (ORFs). Two major polyproteins (pp) are encoded in ORFs, 1a (nsp1-nsp10) and 1ab (nsp11-nsp16), the processed proteins of which make up the replication/transcription complex. pp1ab is synthesized *via* a translation frameshifting (denoted FS) mechanism. The two virally encoded proteases PLPro (papain-like, black) and 3C-like main protease (MPro, red) are responsible for processing pp1a and pp1ab. In the precursor form, MPro is suggested to be anchored on either side with membrane-spanning helices (white bars) within nsp4 and nsp6. MPro is responsible for its own release (termed autoprocessing) and cleavage of the rest of the sites between nsp4 and nsp16. **B** Expression of MPro^D48Y/ΔP168^-GP-6H. Uninduced (lane 1). Distribution of MPro^D48Y/ΔP168^-GP-6H in the soluble (lane 2) and insoluble (lane 3) fractions quantified by subjecting cells (25 ml) to lysis followed by fractionation of the soluble and insoluble fractions and small-scale nickel-affinity chromatography (see “Methods” section for details). **C** Purified MPro^D48Y/ΔP168^ dimer. **D** N-terminal autoprocessing of ^(−102)^MPro^D48Y/ΔP168^-GP-6H precursor (46.5 kDa) in *E. coli* results in MPro ^D48Y/ΔP168^-GP-6H (34.7 kDa) and 11.8 kDa products. Only the 34.7 kDa product is retained in the column because of the 6H tag being at the C-terminus. The appearance of the 11.8 kDa upon cleavage was verified in an earlier work using a similar precursor construct (see Fig. 3a, c in ref. ^6^). Cells (12 ml) were harvested at the indicated time points, and equal volumes of the bound fractions following nickel-affinity chromatography (NAC) were analyzed by SDS-PAGE. Proteins were subjected to SDS-PAGE on 4–20% gradient gels. M denotes protein standards in kDa.

A single copy of SARS-CoV-2 main protease (MPro, nsp5) is encoded within large polyproteins (pp1a and pp1ab), which encompass all nonstructural proteins (nsps) 1–10 and 1–16, respectively [Fig. 1^1–3^]. MPro mediates its own release, termed autoprocessing, through stepwise cleavages at its termini, accompanied by stable dimer formation and appearance of mature-like catalytic activity^4–7^. Processing of the polyproteins to mature nsps is critical for virus assembly and production of viable progeny virions^8^. Therefore, the development of various small molecule antivirals that bind competitively to the active site of mature MPro either covalently or noncovalently and elicit inhibition of its catalytic activity has been remarkably effective to restrict the progression of COVID-19^9^, together with other salient strategies aimed at virus neutralization and entry as well as perturbing different stages of the viral life cycle by targeting other key functional components of the virus^10^.

The chymotrypsin-like fold of the MPro catalytic region (residues 10–185) is connected to a helical region (domain III, residues 201–306) through a long loop (residues 186–200) [Fig. 1^11,12^]. Transient intra- and intermolecular N-terminal autoprocessing of the nsp4/nsp5 cleavage site governs the initial events of MPro release from its precursor^5,6,13–15^. Stable dimer formation is concomitant with the reorganization of the free N-terminal residues, Ser1 residue capping the substrate binding S1 subsite of the opposite protomer through an interaction with E166, G11 mediated interface, as well as R4 and M6 interactions with the helical domain residues E290 and R298, respectively^15–18^. Accordingly, G11A, E290A and R298A mutations lead to increases in the dimer dissociation constant of mature MPro^13,15,18^. The latter two contacts, presumed to occur in conjunction with the repositioning of domain III orientation to promote an inter-domain III interface formed by residues 280 and 283 to 286^11,12,19^ and thereby collectively restrict the motion of domain III in the dimer conformation, appear to be essential for C-terminal autoprocessing, i.e., nsp5/nsp6 cleavage^6,20^. Importantly, all of the above conformational changes are tightly coupled to the active site oxyanion loop equilibrium transitioning from an inactive unwound conformation (E-state) of the monomer to an active wound conformation (E*-state), typical of the mature fully active dimer^14,18^. Recent structural studies indicate that the E* to E shift initiates from G138-F140 residues, followed by the oxyanion hole unwinding^15^.

Emergence of viral variants and drug resistance to known therapies for the treatment of SARS-CoV-2 infections is well documented^10^. Therefore, understanding the molecular basis of drug resistance is vital to the development of future compounds to limit the progression of viral variants, as well as providing insights into combination drug therapy. Our focus has been on understanding how selected mutations alter critical features of MPro regulation and function, such as autoprocessing, dimerization, and catalytic activity, and how to relate those changes to the efficacy of binding of potent inhibitors^21^.

Of all the natural variants described, there is one outlier which involves a deletion mutation of P168 (ΔP168)^22–24^. P168S substitution mutation and ΔP168 account for 76% and 21%, respectively, of changes at this position^24^. In this study, we focus exclusively on a double mutant MPro^D48Y/ΔP168^ described by the Harris group^24^ showing ~50-fold resistance to the clinical drugs, covalent nirmatrelvir (NMV^25^) and noncovalent ensitrelvir (ESV^26^), using a live-cell gain-of-signal assay ^27^. Individually, D48Y shows ~2 and 5-fold, and ΔP168, 5.1 and 6.8-fold resistance to NMV and ESV, respectively ^24^.

First, we optimize the conditions for the expression of the double mutant, which otherwise expresses poorly in normal growth and expression conditions in Luria-Bertani (LB) medium. N-terminal autoprocessing of a MPro^D48Y/ΔP168^ model precursor in *E. coli* was examined. Next, the dimer dissociation constant (*K*_dimer_) and catalytic activity of mature MPro^D48Y/ΔP168^ were determined for comparison with MPro^WT^. Inhibitor dissociation constants and binding thermodynamics of three covalent [GC373, NMV, and pomotrelvir (PMV)^28^] and one noncovalent (ESV) inhibitors to MPro^D48Y/ΔP168^ were assessed by isothermal titration calorimetry (ITC). To evaluate the effects of the mutations on the structure of MPro and binding of these inhibitors, X-ray crystal structures of MPro^D48Y/ΔP168^ in the inhibitor-free (apo) and inhibitor-bound forms were determined at room temperature. Crystallizing the inhibitor-free form of MPro^D48Y/ΔP168^ allowed inhibitor soaking to obtain crystal structures of the corresponding complexes, MPro^D48Y/ΔP168^-GC373, MPro^D48Y/ΔP168^-NMV, MPro^D48Y/ΔP168^-PMV, and MPro^D48Y/ΔP168^-ESV. None of the inhibitor-bound complexes produced crystals by co-crystallization. Conversely, we were successful in growing crystals of MPro^WT^-ESV by co-crystallization and determined the room-temperature structure of the complex to allow for a direct comparison with the MPro^D48Y/ΔP168^-ESV complex. Although cryo-X-ray structures of MPro^WT^-ESV have been reported previously ^26,29–31^, some of these structures contain inaccuracies in the structural refinements, as described elsewhere^15,21^. Molecular dynamics (MD) simulations were carried out to identify differences among the inhibitor-free and bound states of the wild type and the mutant enzyme and related to observed conformational changes of the active site and parameters that govern dimerization and catalytic efficiency.

## Results

### Production of mature MPro^D48Y/ΔP168^ dimer for biochemical and structural studies

Growth and induction in LB medium at 37 °C of the construct MPro^D48Y/ΔP168^-GP-6H (Fig. S1) without a nonnative tag^32,33^ or native nsp4 residues (Fig. S1^6^,) appended to the N-terminus of MPro, yields very low accumulation of intact soluble MPro^D48Y/ΔP168^ like that also observed for a similar construct of MPro^WT^. This was overcome by growth and induction in MagicMedium at 18 °C for 16–18 h, yielding >25-fold more protein (~7 mg/300 ml culture) than that observed in LB medium. The majority of the expressed protein accumulating in the soluble fraction (Fig. 1B) was purified, including the removal of the GP-6H tag using HRV protease. MPro^D48Y/ΔP168^ (Fig. 1C) was verified by mass spectrometry and used for studies described below.

### N-terminal autoprocessing of ^(−102)^MPro^D48Y/ΔP168^ precursor

Release of the free N-terminus upon N-terminal autoprocessing promotes stable dimer formation through interactions of N-finger residues, making crucial intra- and intermonomer contacts with the catalytic region and domain III. Therefore, a construct like that used to evaluate N-terminal autoprocessing of MPro^WT^ and drug-resistant mutants^21^, was engineered containing 102 amino acids of nsp4 (−102) fused to the N-terminus of MPro^D48Y/ΔP168^ (Figs. 1 and S1). Details of the expression and small-scale purification of ^(−102)^MPro^D48Y/ΔP168^-GP-6H precursor are provided in the “Methods” section. Residue P of GP precludes C-terminal autoprocessing^32^, and thereby enables isolation of the MPro^D48Y/ΔP168^-GP-6H product to solely evaluate N-terminal autoprocessing at the nsp4/nsp5 site. In a control experiment, an active site C145A mutation of the wild-type precursor was shown to abolish autoprocessing^21^. Clearly, ^(−102)^MPro^D48Y/ΔP168^ undergoes N-terminal autoprocessing efficiently, like that of ^(−102)^MPro^WT^^21^, with the precursor converting to mature double mutant within 45 min of expression (Fig. 1D). The slightly decreased catalytic activity and decreased *K*_dimer_ of the double mutant (see below) appear not to alter N-terminal autoprocessing in *E.coli*. Comparison of shorter induction times also results in observing the majority of the precursor converted to mature MPro^D48Y/ΔP168^-GP-6H similar to that of the wild type precursor (See Supporting Information in ref. ^21^). Thus, both mutations do not markedly affect the early step in the MPro regulation to the same extent as mutations of critical dimer interface residues^6,14^, which significantly retard autoprocessing. Similar in strategy to the first construct, the processed mature MPro^D48Y/ΔP168^-GP-6H provides another source for the purification of the double mutant protein as described for other drug-resistant mutants^21^.

### *K*_dimer_ and catalytic activity of MPro^D48Y/ΔP168^

**Fig. 2:**
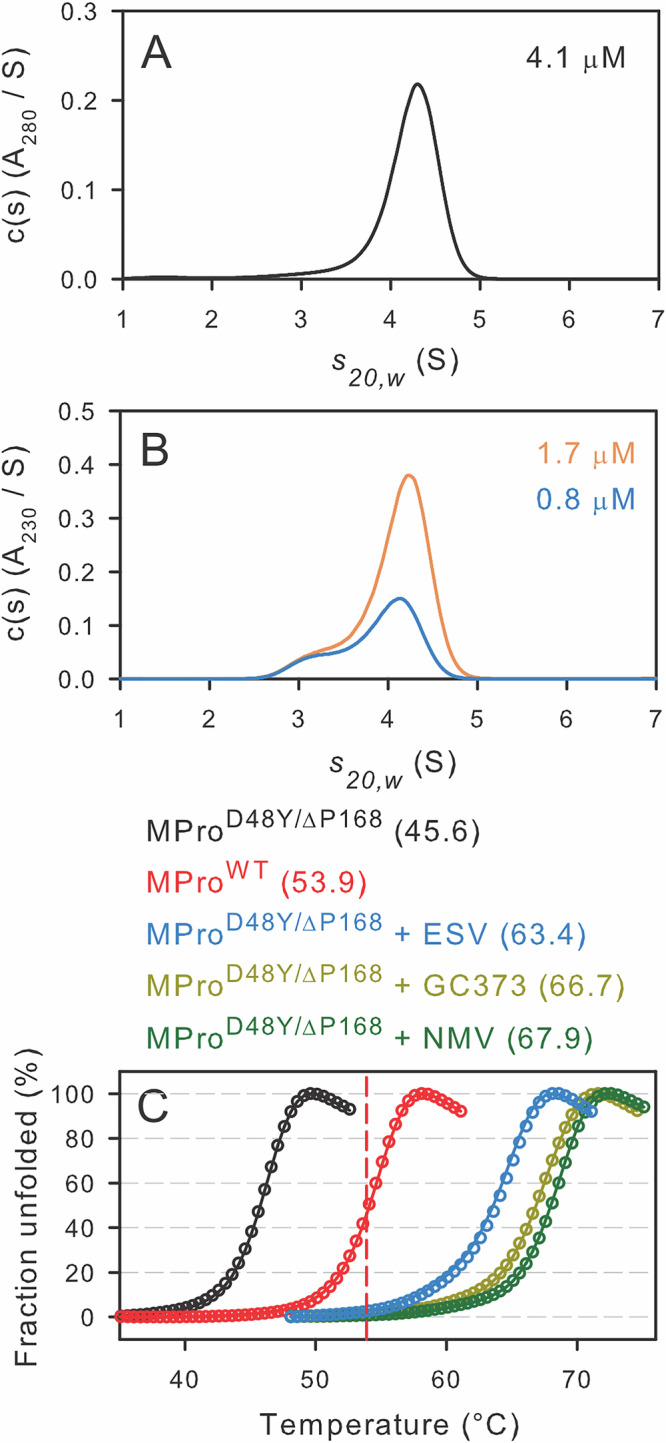
Concentration distribution of sedimentation coefficients c(s) in absorbance units and DSF profiles of mature MPro^D48Y/ΔP168^. Sedimentation velocity profiles at (**A**) 4.1 µM and (**B**) 1.7 µM (orange) and 0.8 µM (blue). Absorbance data in (**A**) were collected at 280 nm, whereas absorbance data in (**B**) were collected at 230 nm. In all cases, 12 mm pathlength cells were used. **C** Thermal denaturation DSF traces at a final concentration of 10 µM protein in the absence and presence of 20-fold molar excess of inhibitor over the protein concentration. Circles and lines indicate duplicate samples. Estimated *T*_m_ midpoints are listed in parentheses beside the construct designation above (**C**). The vertical dashed red line indicates the midpoint for MPro^WT^ transition and serves as a reference point to compare the other traces.

MPro^D48Y/ΔP168^ was subjected to sedimentation velocity analytical ultracentrifugation (SV-AUC) and Lamm equation modeling to estimate the *K*_dimer_ for comparison with MPro^WT^. The estimated *K*_dimer_ of SARS-CoV and SARS-CoV-2 MPro^WT^ using a similar method is ~2.5 µM^33^ and used as a reference point for comparison. Surprisingly, the *K*_dimer_ of MPro^D48Y/ΔP168^ is ~40-fold lower than MPro^WT^ with an estimated value of 0.06 ± 0.01 µM (Figs. 2A, B and S2). Estimation of the *K*_dimer_ allowed calculating the *k*_cat_/*K*_m_ of the double mutant, similar to the enzyme kinetics recently described for the drug-resistant mutants^21^. At a substrate concentration of 50 µM, good first-order kinetics are observed (Fig. S3), indicating that *K*_m _>> [S]. The calculated *k*_cat_/*K*_m_ of 1.52 ± 0.02 µM^−1^ min^−1^ of MPro^D48Y/ΔP168^ is nearly identical to that of the single mutant MPro^L50F^ and comparable to that of MPro^WT^ (*k*_cat_/*K*_m_ = 2.41 ± 0.02 µM^−1^ min^−1^^21^).

### Thermal stability of MPro^D48Y/ΔP168^ and its resistance to unfolding upon inhibitor binding

Differential scanning fluorimetry (DSF) profiles of MPro^WT^ and MPro^D48Y/ΔP168^ in the absence and presence of inhibitors are plotted (Fig. 2C), and the corresponding *T*_m_ values are listed above the plots. Surprisingly, MPro^D48Y/ΔP168^ shows an 8.3 °C decrease in *T*_m_, relative to MPro^WT^. The difference in *T*_m_ of the inhibitor-free and bound complexes (Δ*T*_m_) of MPro^D48Y/ΔP168^ as compared to corresponding MPro^WT^ samples are listed in Table 1^15,34^. Inhibitor binding increases resistance to unfolding (Δ*T*_m_) in the range of 18–21 °C, which relates to the strength of binding, and can be correlated with the measured *K*_d_ determined by ITC. The slightly lower Δ*T*_m_ observed for MPro/ESV complexes, even though the *K*_d_ for ESV binding is lower, may relate to it being a noncovalent binder as compared to the covalent binding mode of NMV and GC373.

**Table 1:** *K*_d_ and thermodynamic parameters of inhibitor binding to MPro^WT^ and MPro^D48Y/ΔP168^

Inhibitor	*K*_d_-fold increase	1/K_a_ (µM)	Δ*H*, kcal/mol	Δ*S*, cal/mol/K	Δ*G*, kcal/mol	Δ*T*_m_ (°C)
MPro^WT^ + GC373	1	0.15 ± 0.03	−6.7 ± 0.10	9.1	−9.4	19.8
MPro^D48Y/ΔP168^ + GC373	0.9	0.14 ± 0.02	−9.1 ± 0.10	1.27	−9.4	21.1
MPro^WT^ + NMV	1	0.007 ± 0.003	–10.8 ± 0.70	1.57	–11.2	21.1
MPro^D48Y/ΔP168^ + NMV	3	0.018 ± 0.009	−9.5 ± 0.1	3.74	−10.7	22.3
MPro^WT^ + ESV	1	0.006 ± 0.003	−15.4 ± 0.01	−13.9	−11.2	18.9
MPro^D48Y/ΔP168^ + ESV	5	0.028 ± 0.007	−12.2 ± 0.01	−6.01	−10.4	17.8

Titrations were carried out in buffer B at 28 °C with 30 µM protein in the cell and inhibitor (in the syringe) at 10 times the protein concentration. Previous ITC results of MPro^WT^ with the corresponding inhibitors are included for ease of comparison^14,18,36,47^. Δ*T*_m_ denotes the difference in *T*_m_ measured in the presence and absence of inhibitor (Fig. 2C).

### Inhibitor dissociation constant (*K*_d_) and binding thermodynamics of covalent and noncovalent inhibitors to MPro^D48Y/ΔP168^

ITC results summarized in Table 1 provide a comparison of the extent of resistance rendered by the mutations D48Y and ΔP168 assessed by in vivo live-cell gain-of-signal assay ^27^ with in vitro estimation of the *K*_d_ by ITC. Binding isotherms of inhibitors to mature MPro^D48Y/ΔP168^ are shown in Fig. S4. Clearly, the single binding isotherms are indicative of the active sites being equivalent in the mutant dimer. The results indicate that the *K*_d_ for the GC373 binding to MPro^D48Y/ΔP168^ is about the same as GC373 binding to MPro^WT^. NMV and ESV, which exhibit ~20-fold lower *K*_d_ than GC373 to MPro^WT^, show 3- and 5-fold decreased affinity to MPro^D48Y/ΔP168^, respectively, relative to their binding affinity to MPro^WT^. Relative to the wild type, the observed small increases in the binding constants with inhibitors parallel a proportional decrease of ~40% in the catalytic efficiency calculated for the double mutant.

Interestingly, the D48Y substitution and P168 deletion mutations exert an increase in the favorable enthalpy change for GC373 binding, which is offset by a decrease in favorable entropy and thereby resulting in a net ΔG that is identical to GC373 binding to MPro^WT^. For NMV binding, which follows similar thermodynamic binding profiles like that of GC373, a small decrease in favorable enthalpy is offset by an increase in favorable entropy. ESV binding to MPro^WT^ exhibits the opposite of GC373 and NMV, resulting in an unfavorable binding entropy and thereby decrease in the net gain in free energy. Interestingly, the decrease in favorable enthalpy is offset by a decrease in unfavorable entropy change for ESV binding to MPro^D48Y/ΔP168^, relative to MPro^WT^. The poor solubility of PMV under the same conditions precluded reliable analysis by ITC.

### Structure and dynamics of inhibitor-free MPro^D48Y/ΔP168^

**Fig. 3:**
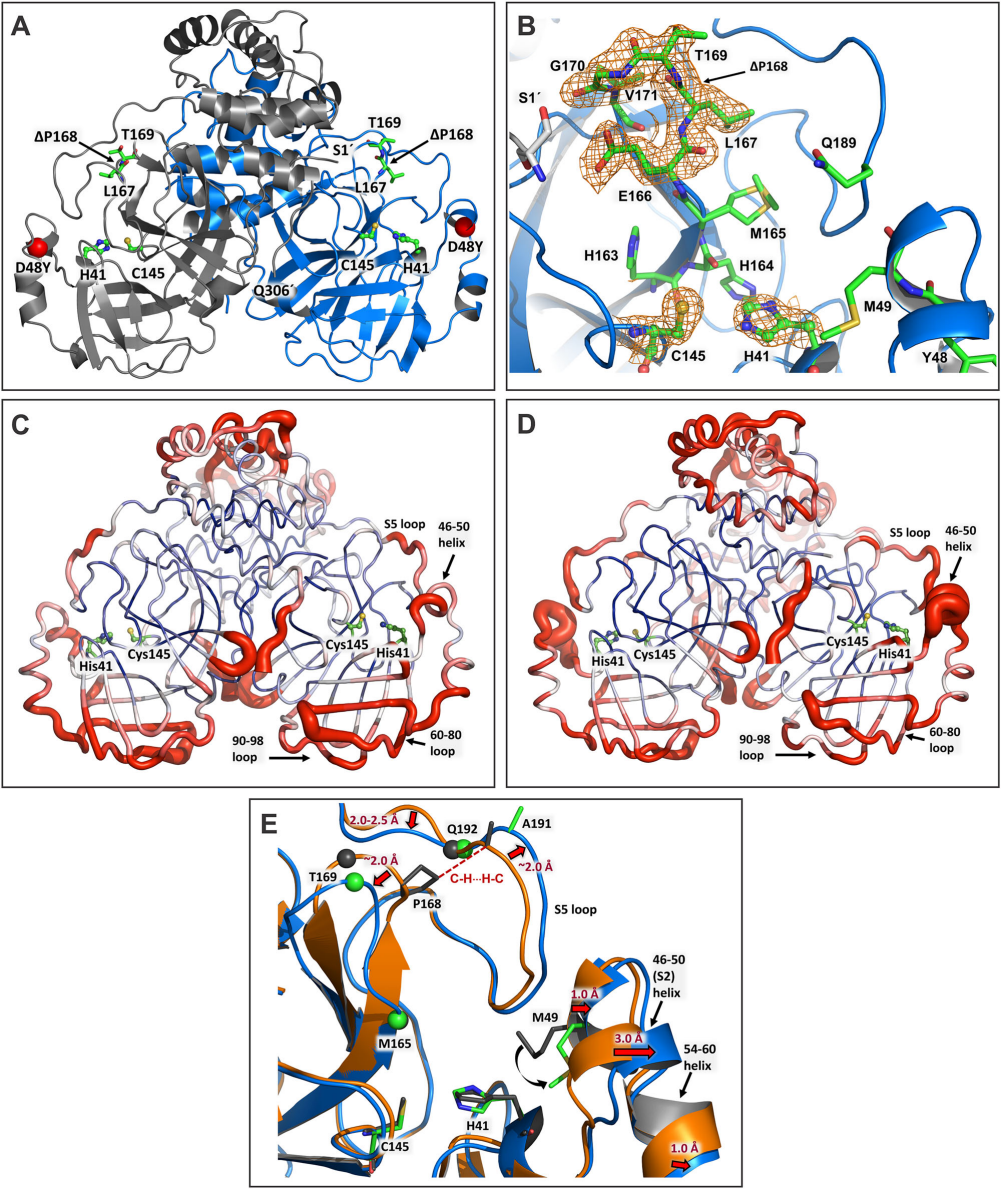
Structure and dynamics of inhibitor-free mature MPro. **A** MPro^D48Y/ΔP168^ double mutant is depicted in cartoon representation showing positions of mutations. One monomer is colored blue and the other is in gray. **B** The catalytic and active site residues in the double mutant. The 2F_O_-F_C_ electron density maps are contoured at 1.5 *σ*. Cartoon-putty representation of the MPro structure colored by averaged backbone atomic displacement parameters (B-factors) for MPro^WT^ in (**C**) (PDB ID 7JUN) and for MPro^D48Y/ΔP168^ in (**D**). Redder and thicker putty depicts higher B-factors. **E** Superimposition of MPro^D48Y/ΔP168^ on MPro^WT^. Green and dark gray spheres show the Cα atom positions of the corresponding residues.

Room-temperature X-ray structure of MPro^D48Y/ΔP168^ was obtained in the inhibitor-free form at 1.85 Å resolution (Table 2). We compare the current double mutant structure with our previous room-temperature X-ray structure of MPro^WT^ [PDB 7JUN^12^,] to reveal the effects of introducing the D48Y substitution and P168 deletion (Fig. 3). MPro^D48Y/ΔP168^ crystals are monoclinic in space group I2 and the unit cell dimensions that are different from those of MPro^WT^, accommodating one enzyme protomer in the asymmetric unit, with the native homodimer generated through the crystallographic two-fold axis. Such crystals were shown previously to be amendable to inhibitor soaking^35,36^, allowing inhibitor complexes to be generated as described below. Residue D48 is part of a short 310-helix spanning residues 46–50 (termed S2 helix henceforth) that creates a side wall for the hydrophobic substrate binding subsite S2, which D48 itself is not part of (Fig. 3A). Adjacent residue M49 participates in building subsite S2 and interacts with the P2 groups of inhibitors. P168 is located at the tip of a β-hairpin loop consisting of residues 167–170, that acts as a lid of subsite S4, binding hydrophobic P4 groups of substrates and inhibitors. The β-hairpin loop is next to the critical E166 residue that pre-organizes subsite S1 and is part of the dimer interface by interacting with the N-terminal Ser1´ of the other protomer in the MPro homodimer^17,36–40^.

**Table 2:** Crystallographic data collection and refinement statistics

	MPro^D48Y/ΔP168^ PDB ID 9N6J	MPro^D48Y/ΔP168^-GC373 PDB ID 9N6L	MPro^D48Y/ΔP168^-NMV PDB ID 9N6M
Data collection:	X-ray (in-house)		
Diffractometer	Rigaku HighFlux, Eiger R 4 M		
Space group	I2	I2	I2
Wavelength (Å)	1.5406	1.5406	1.5406
Cell dimensions:			
*a*, *b*, *c* (Å)	52.53, 82.59, 91.32	52.77, 81.90, 91.69	52.48, 82.47, 91.37
α, β, γ (°)	90, 95.3, 90	90, 95.3, 90	90, 95.4, 90
Resolution (Å)	61.1–1.85 (1.92–1.85)	60.9 – 2.20 (2.28 – 2.20)	61.1–2.00 (2.07–2.00)
No. reflections unique	32946 (3264)	19382 (1906)	26247 (2629)
*R*_merge_	0.113 (0.702)	0.082 (0.274)	0.102 (0.618)
*CC*_*1/2*_	0.919 (0.535)	0.983 (0.987)	0.951 (0.670)
*<I*/σ*I*>	13.2 (0.97)	24.7 (4.7)	14.2 (1.7)
Completeness (%)	99.3 (98.2)	97.9 (96.3)	99.9 (99.8)
Redundancy	4.5 (4.5)	4.6 (4.7)	5.5 (5.3)
**Refinement:**			
*R*_work_/*R*_free_	0.1897/0.2207	0.1806/0.2243	0.1779/0.2211
*B*-factors			
Protein	40.1	32.3	38.9
Ligand	N/A	39.9	46.1
Water	41.6	31.5	37.3
Inhibitor occupancy	N/A	1.00	0.68
R.M.S. deviations			
Bond lengths (Å)	0.007	0.008	0.009
Bond angles (°)	0.859	0.879	0.970
All atom clash score	2.54	2.98	4.36
Ramachandran stats. (%)			
Favored	97.03	97.69	96.37
Allowed	2.97	2.31	3.63
Outliers	0	0	0

	MPro^D48Y/ΔP168^-PMV PDB ID 9N6N	MPro^D48Y/ΔP168^-ESV PDB ID 9N6P	MPro^WT^-ESV PDB ID 9N6R
Data collection:	X-ray (in-house)		
Diffractometer	Rigaku HighFlux, Eiger R 4 M		
Space group	I2	I2	I2
Wavelength (Å)	1.5406	1.5406	1.5406
Cell dimensions:			
*a*, *b*, *c* (Å)	52.34, 82.37, 91.09	52.26, 83.33, 91.00	52.02, 83.30, 90.97
α, β, γ (°)	90, 95.4, 90	90, 95.5, 90	90, 95.4, 90
Resolution (Å)	61.0–1.85 (1.92–1.85)	61.3–1.90 (1.97–1.90)	61.3–1.70 (1.76–1.70)
No. reflections unique	32821 (3285)	29235 (2850)	42304 (4186)
*R*_merge_	0.102 (0.569)	0.115 (0.544)	0.071 (0.610)
*CC*_*1/2*_	0.954 (0.674)	0.940 (0.774)	0.968 (0.722)
*<I*/σ*I*>	18.9 (1.5)	13.2 (1.9)	22.3 (1.7)
Completeness (%)	99.9 (98.8)	95.4 (93.0)	99.6 (98.4)
Redundancy	4.3 (3.3)	4.7 (4.8)	5.4 (5.2)
**Refinement:**			
*R*_work_/*R*_free_	0.1737/0.2179	0.1679/0.1993	0.1708/0.2020
*B*-factors			
Protein	41.2	37.9	34.1
Ligand	59.7	37.5	30.0
Water	41.6	41.2	37.6
Inhibitor occupancy	0.65	0.75	1.00
R.M.S. deviations			
Bond lengths (Å)	0.011	0.017	0.017
Bond angles (°)	1.197	1.462	1.474
All atom clash score	5.44	4.02	2.12
Ramachandran stats. (%)			
Favored	97.36	97.99	97.33
Allowed	2.64	2.01	2.67
Outliers	0	0	0

Data reduction and refinement statistics for the room temperature X-ray crystal structures of SARS-CoV-2 MPro. Values in parentheses are for the highest-resolution shell.

In MPro^D48Y/ΔP168^, the catalytic C145 and H41 and other active site residues including the deletion site ΔP168 are well ordered as indicated by the strong electron density maps (Fig. 3B). In contrast, the electron density is weak for the S2 helix where mutation D48Y is present, with high atomic displacement parameters (B-factors) for these residues. The B-factor analysis of MPro^WT^ (PDB ID 7JUN) and MPro^D48Y/ΔP168^ room-temperature structures (Fig. 3C, D) demonstrates that the mutant is significantly more dynamic near the sites of mutations, including S2 helix residues 46–50, the S5 loop connecting the catalytic and the helical domains (residues 188–199), and for the entire helical domain. Conversely, surface loops containing residues 60–80 and 90–98 are slightly more rigid in MPro^D48Y/ΔP168^, which cannot be attributed to crystal packing as the closest symmetry-related molecule is over 10 Å away. Our B-factor analysis is in agreement with the multitemperature crystallographic data on Mpro^WT^ reported elsewhere^41^. MPro^D48Y/ΔP168^ superimposes on MPro^WT^ (PDB ID 7JUN) with an RMSD of 0.6 Å, indicating that overall, the structures are similar, but differences are possible for specific regions of the protein structure. Indeed, there are significant shifts of the secondary structure elements in the active site. The active site in MPro^D48Y/ΔP168^ is more open than in MPro^WT^ caused by the movements of residues belonging to subsites S2–S5 (Fig. 3E). Specifically, the helix, spanning residues 46–50, shifts by as much as 3 Å from its position in MPro^WT^ away from the catalytic site, resulting in the adjacent α-helix, which is not part of the active site, containing residues 54–60 to move in unison by about 1 Å. P168 deletion in MPro^D48Y/ΔP168^ results in the β-hairpin loop shortening and a loss of hydrophobic interactions made by P168 with A191. Interestingly, as a consequence, the S5 loop (residues 188–199) shifts in a zig-zag fashion relative to the β-hairpin loop, with residues 188–191 moving away by ~2 Å, Q192 remaining in place stabilized through an H-bonding bridge with the main chain atoms of V186, and residues 193–196 moving towards L167 and T169 by 2–2.5 Å. Overall, these structural changes in MPro^D48Y/ΔP168^ relative to MPro^WT^ lead to the opening of S2 subsite by ~1–3 Å due to the S2 helix shift (measured from residues 46–50 to M165) and M49 side chain conformational change, and the subsites S4/S5 by ~4 Å (measured between P168 or T169 and A191). The dimer interface contacts are similar in both proteins. The only noteworthy difference arises from shorter, thus perhaps stronger, H bonds between the side chains of R4 and Q299 with K137´ and S139´, respectively. This may be a consequence of the oxyanion loop (residues 138–145) shifting slightly by 0.5–0.7 Å in MPro^D48Y/ΔP168^ towards the other protomer (Fig. S5). Stronger interactions between the protomers in the double mutant agree with its improved dimer stability demonstrated by a decreased *K*_dimer_ relative to MPro^WT^.

To compare the protein dynamics of MPro^WT^ and MPro^D48Y/ΔP168^, we performed 1 μs simulations of the corresponding homodimers, assigning the protonation states to the ionizable residues according to the observed hydrogen atom positions in our neutron structure of MPro^WT^ (Fig. S6A, B)^12^. Importantly, the sub-microsecond dynamics of the protomers are not identical (asymmetric) in the MD simulations, although crystal packing makes the MPro protomer structures identical. This dynamic behavior of Mpro^WT^ has been previously observed in molecular simulations^42^. In agreement with the B-factor analysis from the X-ray structures, the root mean square fluctuation (RMSF) analysis shows that the S2 helix is more dynamic while the region with residues 60–100 is less dynamic in MPro^D48Y/ΔP168^ in one of the protomers, compared to that in MPro^WT^ (Fig. S6C, D). Similarly, the helical domain, including the C-terminal tail is slightly more dynamic in the double mutant in both protomers. In addition, the active site openness, calculated as the distance between residue 47 of the short subsite S2 helix and residue 169 at the tip of the subsite S4 β-hairpin loop, fluctuates on the sub-microsecond scale for both MPro^WT^ and MPro^D48Y/ΔP168^ between more open and more closed conformations relative to the starting models made from the X-ray structures to settle in more closed conformations that are similar for both proteins in the range of 900 ns to 1 μs (Fig. S6E, F). Although these results are consistent with the B-factor analysis indicating that MPro^D48Y/ΔP168^ is more dynamic than MPro^WT^, we emphasize here that the current MD simulations do not probe much slower dynamics on the microsecond-to-millisecond timescale, which may also be important for the protein unfolding measured by DSF.

### Binding of covalent inhibitors to MPro^D48Y/ΔP168^ and comparison with MPro^WT^ complexes

To analyze the structural ramifications of the mutations in MPro^D48Y/ΔP168^ on the binding of covalent inhibitors GC373, NMV, and PMV in comparison with MPro^WT^, we obtained room-temperature X-ray crystal structures of MPro^D48Y/ΔP168^-GC373, MPro^D48Y/ΔP168^-NMV, MPro^D48Y/ΔP168^-PMV complexes at resolutions of 1.85–2.2 Å (Table 2 and Fig. S7). GC373 was found at 100% occupancy in the mutant active site, whereas NMV and PMV showed occupancies of 68% and 65%, respectively, which may be attributed to the fact that the complexes were obtained by soaking the inhibitors into the inhibitor-free crystals of MPro^D48Y/ΔP168^. All atoms in each inhibitor were refined to have the same occupancy and equal to the overall inhibitor occupancy. For structural comparison, we use the room-temperature X-ray structures of MPro^WT^-GC373 [PDB ID 7UKK^14^] and MPro^WT^-NMV [PDB ID 7SI9^36^], and a cryo-temperature X-ray structure of MPro^WT^-PMV [PDB ID 8TBE^28^]. GC373 binding affinity is virtually the same, whereas that of NMV decreased slightly by about three-fold compared to the values for MPro^WT^, indicating minimal effect of the mutations on the binding of the covalent inhibitors. The binding affinity of PMV could not be measured due to poor inhibitor solubility, as discussed above.

**Fig. 4:**
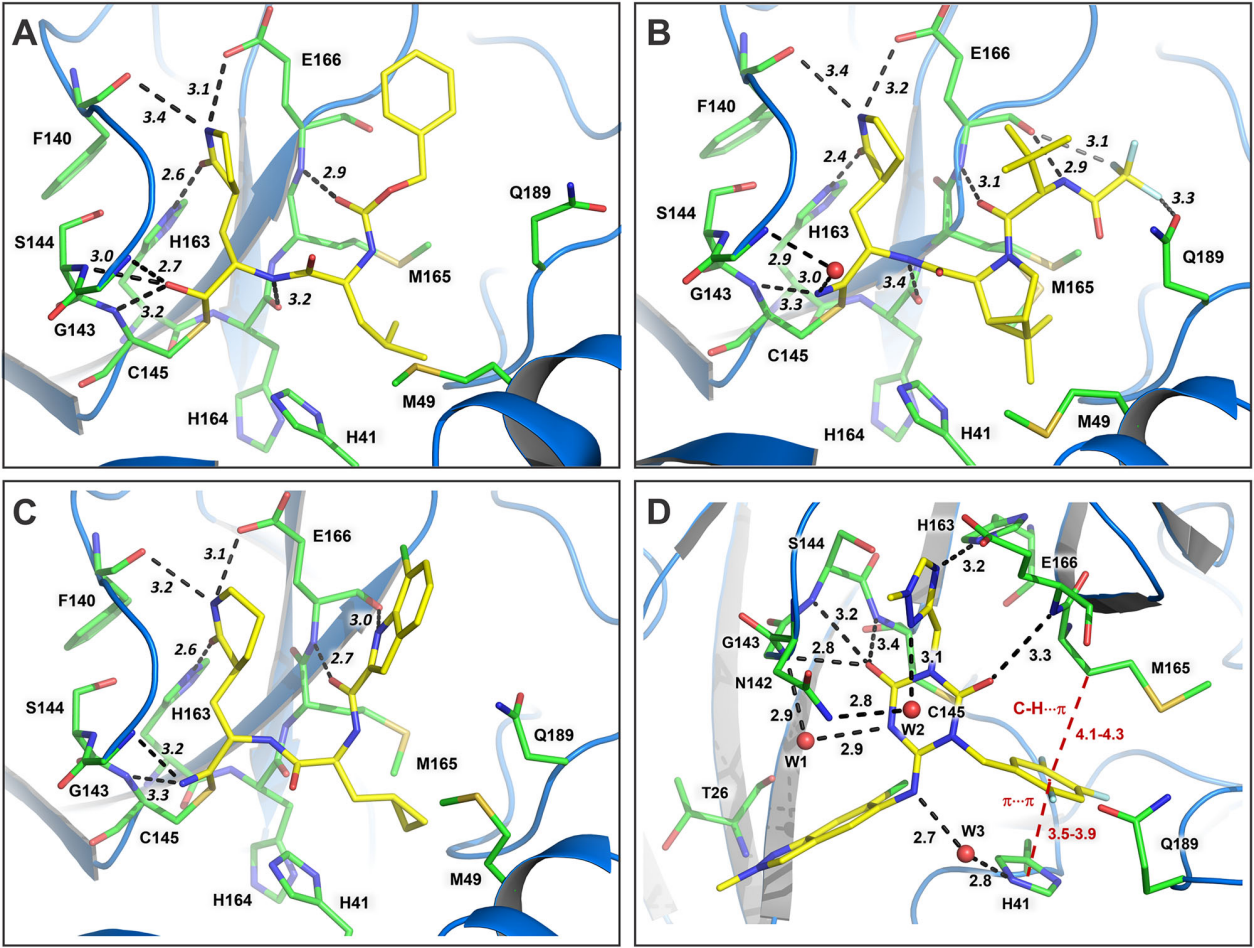
Intermolecular interactions of covalent and noncovalent inhibitors with MPro^D48Y/ΔP168^. **A** Complex with GC373. **B** Complex with NMV. **C** Complex with PMV. **D** Complex with ESV. Inhibitors are depicted with carbon atoms colored yellow, and the double mutant residues have carbon atoms colored green. All distances are shown in Angstrom.

The electron density maps for GC373, NMV, and PMV bound to the active site of MPro^D48Y/ΔP168^ are shown in Fig. S8A–C. GC373’s aldehyde warhead reacts with the C145 nucleophilic thiol to generate the reversible hemithioacetal covalently linking the inhibitor to the mutant. NMV and PMV both have nitrile warheads that upon reaction with C145 are converted into thioimidate esters, the covalent linkage shown previously was stable for isolation and characterization for NMV^14^. The hydroxyl of GC373 hemithioacetal points into the oxyanion hole making H bonds with the main chain amide NH groups of G143, S144 and C145 (Fig. 4A). The inhibitor is further stabilized in the double mutant’s active site by H bonds with the side chains of H163 and E166, and with the main chain atoms of F140, H164 and E166. Similarly, the nitrogen of the thioimidate ester is directed into the oxyanion hole in MPro^D48Y/ΔP168^-NMV and MPro^D48Y/ΔP168^-PMV complexes, but, unlike the hemiacetal hydroxyl of GC373, the thioimidate nitrogen makes weak H bonds. In the double mutant complex with NMV, the thioimidate nitrogen forms one direct 3.3 Å H bond with the main chain of C145, whereas in the PMV complex, there are two direct H bonds with C145 and G143 with the distances of 3.2 Å and 3.3 Å, respectively (Fig. 4B, C). In MPro^D48Y/ΔP168^-NMV, a direct H bond with G143 seen for PMV thioimidate nitrogen, is replaced with a water-mediated interaction. NMV and PMV interact with the double mutant through additional H bonds with the H163 side chain, F140 main chain, and with both the main chain and side chain atoms of E166. NMV also makes unconventional C-F···O contacts with the E166 main chain carbonyl and Q189 side chain amide. Surprisingly, based on the B-factor analysis, the binding of covalent inhibitors did not noticeably reduce the dynamic features of the active site regions near the inhibitors nor for the helical domains in these complexes demonstrating that the double mutant remains dynamic when inhibited (Fig. S9A–C) even when the *K*_dimer_ is 40-fold lower than MPro^WT^.

**Fig. 5:**
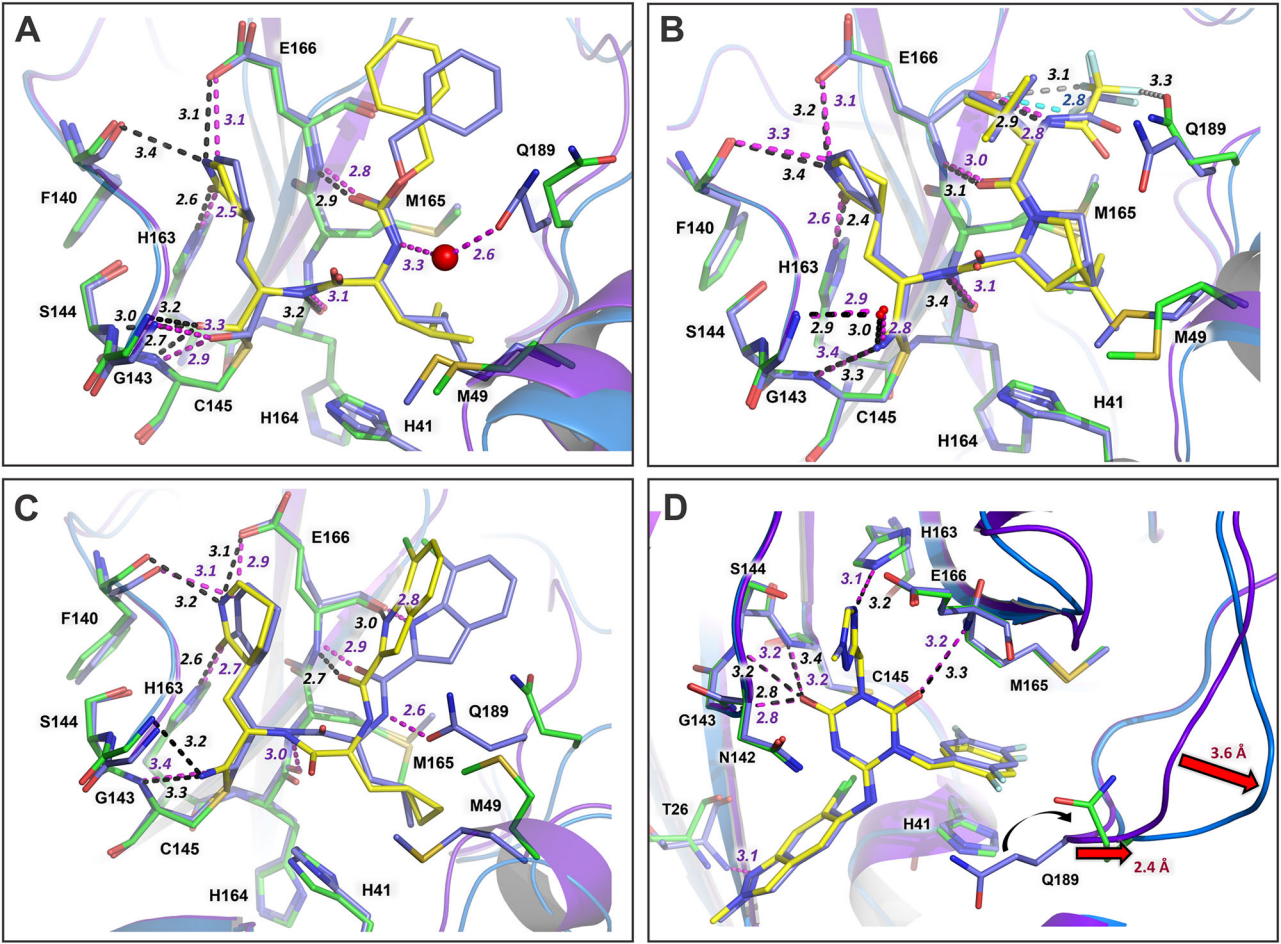
Superpositions of the double mutant inhibitor complexes on the corresponding MPro^WT^ complexes. **A** MPro^D48Y/ΔP168^-GC373 aligned with MPro^WT^-GC373 (PDB ID 7UKK). **B** MPro^D48Y/ΔP168^-NMV aligned with MPro^WT^-NMV (PDB ID 7SI9). **C** MPro^D48Y/ΔP168^-PMV aligned with MPro^WT^-PMV (PDB ID 8TBE). **D** Superimposition of the MPro^D48Y/ΔP168^-ESV and MPro^WT^-ESV complexes. Double mutant carbon atoms are colored yellow and green and cartoon in blue; the MPro^WT^ carbon atoms are purple with a cartoon model in light purple. All distances are in Angstrom.

As anticipated, GC373 binding to the double mutant is identical to the inhibitor’s binding in MPro^WT^. MPro^D48Y/ΔP168^-GC373 complex superimposes onto MPro^WT^-GC373 structure (PDB ID 7UKK) with the RMSD of 0.4 Å on the main chain atoms. H bonds formed by the inhibitor are mostly not altered by the mutations and their distances do not differ by more than 0.1 Å in the double mutant structure compared to MPro^WT^-GC373 complex, except for the hemithioacetal that appears to bind tighter to MPro^D48Y/ΔP168^ oxyanion hole where H bonding distances shorten by 0.2–0.3 Å (Fig. 5A). Conversely, GC373 loses a water-mediated contact with Q189 in the double mutant, where this residue’s side chain rotates away from the inhibitor accompanied by a shift of residues 189–191 by ~1 Å in the same direction. In addition, the S2 helix residues 46–50 move away from the GC373 P2 substituent by 1.3–1.7 Å, reducing hydrophobic interactions made by M49. It appears that the interplay of the strengthened H bonds compensates for the weakened hydrophobic interactions of GC373 with the MPro^D48Y/ΔP168^ active site, leading to the observed unchanged inhibitor binding affinity relative to MPro^WT^ as measured by ITC.

MPro^D48Y/ΔP168^-NMV complex superimposes onto MPro^WT^-NMV structure (PDB ID 7SI9) with the RMSD of 0.3 Å on the main chain atoms, indicating the structures are almost identical. Like GC373, NMV forms similar H bonds with the double mutant compared to MPro^WT^, except the H bond with the main chain carbonyl of H164 is elongated and the P4 CF3 group is bound less tightly than in the wild type enzyme active site (Fig. 5B). Also, as in MPro^D48Y/ΔP168^-GC373 complex, the S5 loop and the S2 helix are shifted by ~1 Å away from NMV in the double mutant reducing the inhibitor’s interactions with the active site. This can explain the slight increase in the NMV’s *K*_d_ value for the double mutant compared to that for MPro^WT^. Remarkably, MPro^D48Y/ΔP168^-PMV and MPro^WT^-PMV (PDB ID 8TBE) structures are the most dissimilar, aligning on the main chain atoms with the RMSD of 0.7 Å (Fig. 5C). Although the H bonding interactions made by PMV are similar in the two structures, the positions of the inhibitor’s P3 substituent and of the double mutant’s S5 loop are significantly different. The dissociation constants of PMV could not be measured by ITC, and thus we cannot evaluate how the two mutations affect PMV binding affinity. The PMV inhibitory constant (*K*_i_) for MPro^WT^ was measured to be 2.7 nM^28^, which is very similar to the *K*_d_ values of NMV and ESV (Table 1).

### Binding of noncovalent inhibitor ESV to MPro^D48Y/ΔP168^ and comparison with MPro^WT^ complex

To evaluate how the mutations D48Y and ΔP168 alter the binding of a noncovalent inhibitor ESV relative to MPro^WT^, we obtained room-temperature X-ray crystal structures of MPro^D48Y/ΔP168^-ESV and MPro^WT^-ESV complexes at resolutions of 1.9 Å and 1.7 Å, respectively (Table 2). ESV occupancy was refined to a value of 75% in the double mutant and to 100% in MPro^WT^ structures. The room-temperature X-ray structure of MPro^WT^-ESV (this study) was done for direct structural comparison with the double mutant-ESV complex. The binding affinity of ESV to MPro^D48Y/ΔP168^ decreased by about 5-fold compared to that for MPro^WT^, as measured by ITC, indicating some effect of the mutations on this inhibitor binding.

The electron density map for ESV bound in the active site of MPro^D48Y/ΔP168^ is shown in Fig. S10A. Because the position of the inhibitor is not fully occupied, there are two alternate conformations for the side chains of the catalytic residues C145 and H41, with one conformation of C145 and H41 having an occupancy matching that of ESV (75%). ESV makes no covalent bonds with the enzyme active site residues, interacting instead through intermolecular contacts. The inhibitor forms five direct H bonds, three of which with the oxyanion hole, and three water-mediated interactions with MPro^D48Y/ΔP168^ amino acid residues (Fig. 4D). Similar to the covalent inhibitors, ESV binding did not affect the dynamics of the double mutant (Fig. S10B). Although MPro^D48Y/ΔP168^-ESV superimposes onto MPro^WT^-ESV complex with a low RMSD of 0.4 Å and the inhibitors occupy essentially the same positions, there are significant deviations in H bonding interactions and the overall conformations of the active sites (Fig. 5D). The central substituted triazinane heterocycle interactions with the oxyanion hole are weaker in the double mutant and the indazole P1´ substituent located in the enzyme’s S1´ subsite lost an H bond with the main chain amide NH of T26 relative to the MPro^WT^ complex. Conversely, the water-mediated contacts ESV makes with H41, N142, and G143, and the hydrophobic π···π stacking and C-H···π interactions with H41 and M165 are retained in the double mutant complex. Interestingly, the S5 loop adopts a more open conformation in MPro^D48Y/ΔP168^-ESV than in MPro^WT^-ESV moving 2.4–3.6 Å away from the inhibitor, a conformation that appears to be a common feature of the double mutant in both the inhibitor-free and inhibitor-bound states.

## Discussion

In this study, we carried out a systematic characterization of a natural variant of SARS-CoV-2 MPro bearing a single substitution (D48Y) and the unusual deletion (ΔP168) mutation (Fig. 3A). D48 is part of a short helix of residues 46–50 covering the S2 subsite through M49 side chain interaction. ΔP168, which contributes to 21% of the mutations observed in this position^24^ is part of the S4 subsite residues forming a β-hairpin stretch and interacting with substrate and inhibitor. Together, these mutations contribute 40 to >100-fold resistance to both clinical inhibitors NMV and ESV as evaluated in cell-based assays^24^.

The double mutant MPro^D48Y/ΔP168^-GP-6H construct, in which the C-terminal residues of MPro followed by GP provide specificity for HRV protease cleavage (Fig. S1) following initial nickel-affinity purification, results in very low expression in LB medium and recovery of only partially pure enzyme. Fortuitously, expression of MPro^D48Y/ΔP168^-GP-6H under conditions described for the MagicMedium at 18 °C yielded an appreciable amount of soluble protein for purification to near homogeneity (Fig. 1B, C). Unlike the very low level of expression of MPro^D48Y/ΔP168^-GP-6H, improved expression of the precursor mimetic ^(−102)^MPro ^D48Y/ΔP168^-GP-6H in LB medium at 37 °C permitted a strict comparison of the initial N-terminal processing reaction in *E. coli*, a critical step for dimer stability and catalytic activity, with that previously described for MPro^WT^ and drug-resistant mutant precursors^21^. Our results indicate slight impairment in the N-terminal autoprocessing of ^(−102)^MPro ^D48Y/ΔP168^-GP-6H precursor, given that majority of the precursor was converted to MPro^D48Y/ΔP168^-GP-6H within 15 min of expression like that of the wild type precursor ^(−102)^MPro^WT^-GP-6H (Fig. 1D^21^). The rapid autoprocessing of the ^(−102)^MPro ^D48Y/ΔP168^-GP-6H precursor precluded its isolation and further characterization. Additionally, the insolubility of the ^(−102)^MPro ^D48Y/ΔP168^-GP-6H precursor upon introducing the interface mutations E290A and R298A (denoted as M below) and expression also precluded in vivo and in vitro studies to investigate the early steps in the N-terminal processing of ^(−102)^MPro ^D48Y/ΔP168^-M-GP-6H precursor predominantly as a monomer, as well as the catalytic activity of mature MPro ^D48Y/ΔP168^-M monomer^6,19^.

Therefore, mutant MPro^D48Y/ΔP168^-GP-6H can be purified in one of two ways, followed by HRV protease cleavage to remove the C-terminal 6H-tag as described (Fig. S1^6,18,21^) to attain the mature mutant MPro^D48Y/ΔP168^ of exactly 305 amino acids. Intriguingly, another study failed in its attempt to purify the single mutant MPro^ΔP168^ when replacing the nsp4 (−102) region with a SUMO-tag appended to just the C-terminal nsp4 residues to facilitate intrinsic N-terminal autoprocessing, attributing their observation possibly to poor solubility or aggregation of the expressed protein. However, a similar strategy that employs a GST-tag was shown to be successful for the isolation of various drug-resistant mutants of MPro^32,43^.

Interestingly, mutations D48Y and ΔP168 decrease the *K*_dimer_ of mature MPro^D48Y/ΔP168^ by ~40-fold, relative to MPro^WT^^33^, estimated by SV-AUC, indicating a more stable homodimer having comparable catalytic activity (*k*_cat_/*K*_m_) to that of MPro^WT^. Measured thermal stability of MPro^D48Y/ΔP168^ by DSF contrasts *K*_dimer_ with a decrease of 8 °C, relative to that of MPro^WT^. The decreased *T*_m_ likely reflects the monomer unfolding transition based on the observation that a predominantly monomeric MPro (MPro^M^) exhibits the same *T*_m_ as that of MPro^WT^^34^. Collectively, these results indicate that D48Y and ΔP168 mutations do not affect N-terminal autoprocessing significantly, and the catalytic efficiency of the resultant mature MPro^D48Y/ΔP168^ despite changes in the *K*_dimer_ and thermal stability. It is worth noting that mutations ΔP168/A173V, which render a large increase in NMV resistance, exert minimal effect on catalytic efficiency ^24^. Of the two mutations in MPro^D48Y/ΔP168^, it appears that the P168 deletion contributes mostly to the decrease in *T*_m_ based on the observation that a deletion mutant MPro^ΔP168^ produces a 5 °C decrease in *T*_m_^43^.

Comparison of inhibitor-free MPro^WT^ and MPro^D48Y/ΔP168^ room-temperature structures indicate significant movements of S2, S4, and S5 subsites (Fig. 3E). These deviations relate to the opening of the active site in MPro^D48Y/ΔP168^, defined based on distances measured from S2 helix to M165 and between T169 and A191, as compared to the closed conformation in inhibitor-free MPro^WT^. A side-by-side comparison of the two structures based on B-factor analysis and MD simulations corroborates with enhanced dynamics of the same regions and of the entire helical region (Fig. 3C, D). The fluctuations occur on the sub-microsecond scale for both proteins between the open and closed conformations. A tilt of ~40° of the helical domain observed for three monomeric structures of SARS-CoV MPro^44–46^, relative to its position in the dimer, would suggest that the helical domain can undergo motions independent of the catalytic region (residues 1–185). Therefore, it appears that the opening of the active site via D48Y and ΔP168 mutations is associated with the enhanced dynamics of the helical domain and the connecting loop region, which forms part of the S5 loop (Fig. 3E).

The dimer interface contacts between the two proteins in the absence of bound inhibitor at ambient temperature are similar, but for shorter H bonds between R4 with K137´ and Q299 with S139´ (Fig. S5), which may account for the ~40-fold decreased *K*_dimer_ of the mutant, relative to that of MPro^WT^. This observation suggests that the *K*_dimer_ is modulated independent of the enhanced dynamical motions of the active site, S5 loop, and the helical domain. Interestingly, the decrease in *T*_m_ by 8 °C of the presumed monomer measured by DSF, mainly attributable to the P168 deletion (ΔP168), contrasts the enhanced dimer formation (*K*_dimer_) of the mutant. It is unclear how a C145A mutation^47^, which increases *T*_m_ by 6.8 °C, and ΔP168, exert opposite effects on thermal stability. These significant differences may relate to variations in the slow dynamics on the timescales of tens to hundreds of microseconds between C145A and ΔP168 mutants that are currently unachievable in molecular simulations.

**Fig. 6:**
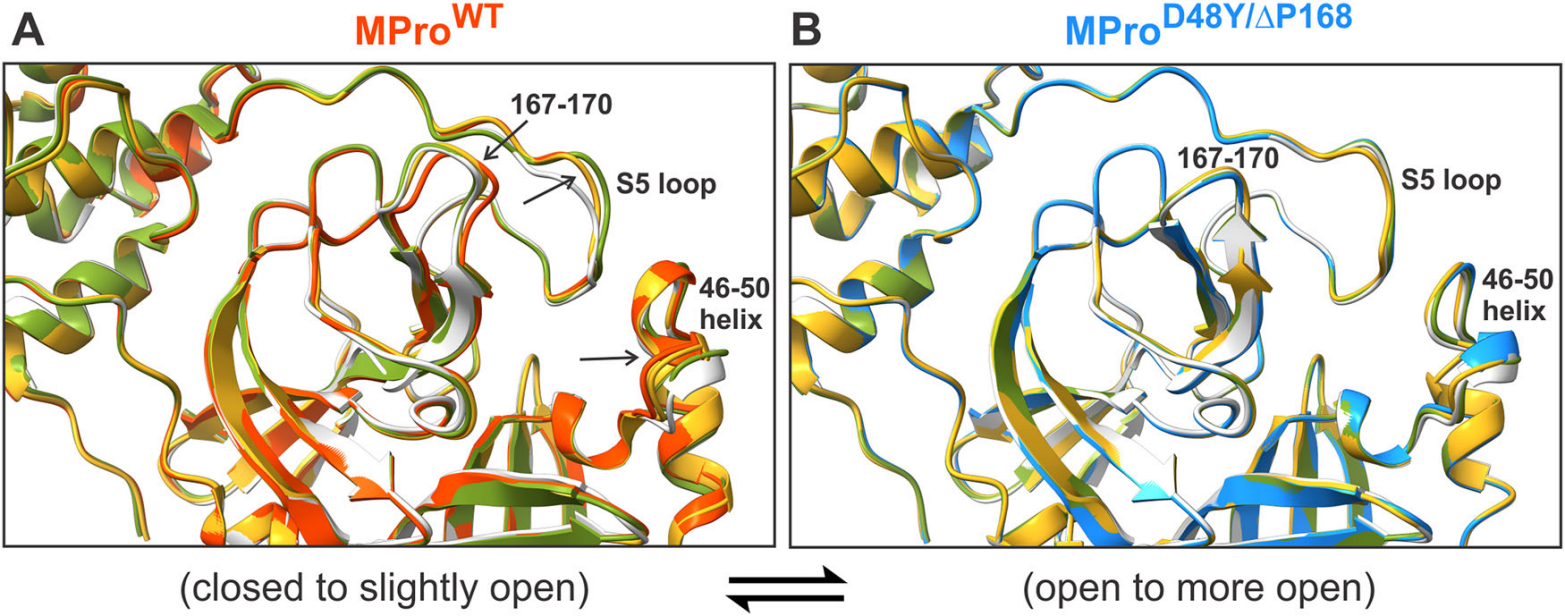
Ribbon overlays of the inhibitor-free and their corresponding inhibitor-bound MPro complexes highlighting overall movements from a closed to a more open active site conformation. Overlays of inhibitor-free MPro^WT^ (**A**, red orange, 7JUN^12^) and double mutant (**B**, dodger blue, this work) on NMV (olive drab), GC373 (goldenrod) and ESV (light gray) bound structures. Compared inhibitor-bound structures in (**A**) are 7SI9^36^ with NMV, 7UKK^14^ with GC373 and 9N6R with ESV (this work). All structures compared in (**B**) are from this work (Table 2). Regions of movement are indicated by black arrows and labels. Distances of the movements comparing the inhibitor-free wild type, and the double mutant are shown in Fig. 3E.

Subtle differences in the binding of the inhibitors contribute to either no change (GC373) or 3- to 5-fold decrease (NMV and ESV, respectively) in the binding affinity. NMV makes an elongated direct H bond with H164 and unconventional contacts through its CF_3_ group, whereas ESV completely loses an H bond with T26 in MPro^D48Y/ΔP168^. Figure 6 highlights the gradual transition from a closed to a more open active site by comparing the inhibitor-free MPro^WT^ and the double mutant with their corresponding inhibitor-bound states. Inhibitor-free MPro^WT^ and the double mutant represent the closed and open state, respectively. Interestingly, inhibitor binding to MPro^WT^ appears to shift the transition to a slightly open state as noted for the 3 regions (Fig. 6A, black arrows and 3E), with MPro^WT^/ESV complex showing the largest change. Since inhibitor-free double mutant is already in an open state, inhibitor binding appears to have little effect on these movements with mutant/ESV complex defined to be in a more open conformation (Fig. 6B). MD simulations and the crystallographic B-factor analysis of the mutant corroborate with this observation suggestive of similar enhanced dynamics for both the inhibitor-free and bound states (Figs. 3, S9 and S10).

It is worth noting that large differences in NMV and ESV resistance observed in cell-based assays^24,27^ are reflected only as small differences in the binding affinity, consistent with only subtle changes in the interaction of the inhibitors with the active site. Collectively, our results suggest that slow and fast dynamics, also potentially influenced by the intracellular environment and molecular crowding events, to offset the effects of mutations, play a critical role in various aspects of the regulation, function, and inhibition of MPro. Aspects such as the effects of molecular crowding on MPro folding dynamics and catalytic activity require future investigations.

## Methods

### Materials

Ni-NTA columns were purchased from Cytiva (Piscataway, New Jersey, USA). His-tagged Human Rhinovirus (HRV) 3C protease was purchased from Sigma (MilliporeSigma, St. Louis, MO). Crystallization reagents and supplies were purchased from Hampton Research (Aliso Viejo, California, USA). Crystallographic supplies for crystal mounting and X-ray diffraction data collection at room temperature were purchased from MiTeGen (Ithaca, New York, USA). GC376 and NMV (or PF-07321332) were purchased from Selleckchem, Houston, TX, and MedChemExpress, NJ, respectively, and ESV was purchased from MilliporeSigma, MA. These compounds are >97% pure by HPLC and NMR analyses, as stated on the vendors’ COA.

### Expression and purification of MPro^D48Y/ΔP168^

Wild type MPro (MPro^WT^) was expressed and purified as described in ref. ^6^. The exact amino acid sequences for preparing purified mature dimer and precursor forms of the variant MPro^D48Y/ΔP168^ are shown in Fig. S1. MPro^D48Y/ΔP168^-GP-6H was synthesized and cloned in pJ414 vector (ATUM, Newark, CA). Precursor ^(−102)^ MPro^D48Y/ΔP168^-GP-6H was synthesized and cloned (Genscript, Piscataway, NJ) into pET11a vector (MilliporeSigma, Burlington, MA) between Nde1 and BamH1 sites. New constructs used in this work and their designations and extinction coefficients are listed in Fig. S1.

Expression of MPro^D48Y/ΔP168^ was enhanced by growth in MagicMedium (Thermo Fisher Scientific, Waltham, MA) to attain sufficient purified MPro^D48Y/ΔP168^ dimer for crystallizations. Cultures in MagicMedium were grown at 37 °C until they reached an OD (600 nm) of 0.8, following which, the temperature was lowered to 18 °C and the cells were harvested after 15–16 h. MPro^D48Y/ΔP168^ dimer was purified from the soluble fraction including the removal of the 6H-tag as described in refs. 6,14,21. A purity of >95% was verified both by SDS-PAGE on 4-20% gradient mini-protean TGX precast gel (Bio-Rad, Hercules, CA) and reverse-phase liquid chromatography with in-line electrospray ionization mass spectrometry^14^.

### N-terminal autoprocessing reaction

Precursor ^(−102)^MPro^D48Y/ΔP168^ was grown in LB medium and induced for expression at 37 °C. Upon induction, 12 ml of culture was drawn at the indicated time point, chilled on ice briefly, and harvested immediately at 4 °C and frozen. Small-scale nickel-affinity chromatography (NAC) was performed by lysis in 500 µl of 50 mM Tris-HCl (pH 8), 20 mM imidazole, 2 mM 2-mercaptoethanol, and 8 M urea. The lysate was spun at full speed in an Eppendorf centrifuge. Derived supernatant was subjected to NAC according to instructions provided for using His SpinTrap (Cytiva, Marlborough, MA) and eluted in 200 µl of lysis buffer containing 500 µM imidazole. Protein concentration was estimated both by Bio-Rad protein assay (Hercules, CA) as well as absorbance at 280 nm. Equal volumes of the bound fraction (6 µl) mixed with the sample buffer and adjusted to a volume of 12 µl were subjected to SDS-PAGE on gels described above, stained with InstantBlue Coomassie Protein stain (abcam, Cambridge, UK) and recorded on a Bio-Rad Gel Doc EZ Imager (Bio-Rad, Hercules, CA). Uncropped gel images of Fig. 1B, C are shown in Fig. S11.

### Enzyme kinetics

Activity assays using the FRET substrate Dabcyl-KTSAVLQ/SGFRKM-E(Edans)-NH_2_, where (/) denotes the scissile peptide bond, were performed in a total volume of 100 µl in buffer A (25 mM Tris-HCl, pH 7.2, 50 mM NaCl, and 1 mM TCEP) at 28 °C as described in refs. ^18,33^. The substrate was custom-synthesized (Biomatik, Ontario, Canada). Reactions containing a final concentration of 0.25 µM and 0.5 µM enzyme and 50 µM substrate were followed by observing the increase in fluorescence^18^. Under these conditions, good first-order kinetics is observed, indicating that *K*_m _>> 50 µM. The observed first-order rate constant (*k*_obs_) is calculated by fitting the exponential rise equation: Y = Y_∞_(1 − e^−kt^) to the entire time course of the reaction using Sigmaplot as described^21^. The k_cat_/K_m_ value for MPro^D48Y/ΔP168^ was calculated as described in our earlier studies of MPro mutants^21^.

### Differential scanning fluorimetry (DSF)

Samples in duplicate were prepared with SYPRO orange dye (5000×, Millipore Sigma product number S5692) to yield a final concentration of 10 µM protein and 5× dye in 25 µl of buffer B (25 mM Tris-HCl, pH 7.2, 20 mM NaCl, and 1 mM TCEP). The fluorescence signal was monitored as a function of temperature in a Bio-Rad C1000 Touch Thermal Cycler, and data were processed with the provided software and plotted using Sigmaplot (Systat Software Inc.). Experiments were repeated at least twice.

### Isothermal titration calorimetry (ITC)

Purified proteins were diluted from a stock solution to slightly above the desired concentration and dialyzed extensively against buffer B (25 mM Tris-HCl, pH 7.2, 20 mM NaCl and 1 mM TCEP). Concentrations were estimated after dialysis based on their 280 nm absorbance at least twice. Stock solutions of inhibitors, GC373 and NMV in buffer B and ESV in DMSO, were diluted in the same buffer to the desired concentration. Titrations were performed with proteins (30 µM) kept in the cell and inhibitors at 10 times the protein concentration in the syringe at 28 °C on iTC200 microcalorimeter (Malvern Instruments Inc., Westborough, MA). Titrations were repeated when needed. Nineteen injections of data were processed using the Origin software provided with the instrument. Raw heat deflections and binding isotherms are shown in Fig. S4.

### Sedimentation velocity analytical ultracentrifugation (SV-AUC)

Samples of MPro^D48Y/ΔP168^ in buffer A at different concentrations loaded in 2-channel centerpiece cells and an An50-Ti rotor were analyzed by SV-AUC. Sedimentation velocity experiments were conducted at 50,000 rpm (201,240 × *g* at 7.2 cm) and 25 °C on a Beckman Coulter ProteomeLab XL-I analytical ultracentrifuge following standard protocols^48^. Sedimentation data were analyzed in SEDFIT^49^ in terms of a continuous c(*s*) distribution of Lamm equation solutions. Solution densities ρ, solution viscosities *η*, and protein partial specific volumes were calculated in SEDNTERP^50^. To estimate the dimer dissociation constants for MPro^D48Y/ΔP168^, absorbance sedimentation velocity data collected at various concentrations were analyzed globally using Lamm equation modeling in SEDPHAT^51^. A monomer-dimer self-association model was used and the presence of both monomer and dimer species was confirmed in the analysis. Absorbance extinction coefficients were calculated in SEDNTERP, and data were plotted in GUSSI^52^.

### Protein crystallization and room-temperature X-ray crystallography

For crystal growth setups, MPro^D48Y/ΔP168^ was concentrated to 5.3 mg/mL and MPro^WT^ was concentrated to 5.5 mg/mL. Inhibitor stock solutions for GC373, NMV, PMV, and ESV were prepared at 50 mM concentrations in 100% dimethyl sulfoxide (DMSO) for crystallization purposes and stored at −30 °C. Inhibitor-free MPro^D48Y/ΔP168^ crystals were grown by sitting drop vapor diffusion methodology with 15–17% PEG3350, 0.1 M Bis-Tris pH 6.5, or 7.0 (1 mL) as the precipitant solution. Crystals of the MPro^WT^-ESV complex were grown by co-crystallization using sitting drop vapor diffusion methodology with 1:10 protein-to-inhibitor molar ratio with the wells containing 16–18% PEG3350, 0.1 M Bis-Tris pH 7.0 (1 mL). Crystallization drops of 20 µL at 1:1 ratio of protein, or protein-inhibitor complex, to well solution were set up using Hampton Research (Aliso Viejo, CA) microbridges and then microseeded as described^36^. Crystals normally appeared after about 7 days and grew to the final size in about 1 month at 14 °C. To prepare complexes of MPro^D48Y/ΔP168^ with GC373, NMV, PMV, and ESV, the inhibitor-free crystals were soaked with the corresponding inhibitor. MPro^D48Y/ΔP168^ crystals were soaked overnight in the crystallization well solutions containing 1:10 molar ratios of GC373, PMV, and ESV, whereas 10-min soaks were performed for NMV to ensure the crystals' integrity. All crystals were mounted in MiTeGen (Ithaca, NY) room-temperature capillary setups for X-ray diffraction data collection.

All room temperature X-ray crystallographic data were collected on a Rigaku HighFlux HomeLab instrument equipped with a MicroMax-007 HF X-ray generator, Osmic VariMax optics, and a DECTRIS Eiger R 4M hybrid photon counting detector. X-ray diffraction data were integrated using the CrysAlis Pro software suite (Rigaku Inc., The Woodlands, TX), then reduced and scaled using Aimless^53^ from the CCP4 suite^54^. Structures were solved by molecular replacement using Phaser^55^. PDB code 7JUN^12^ was used as a search model to solve the inhibitor-free and inhibitor-bound structures. Each model was iteratively refined with phenix.refine from the PHENIX suite^56,57^ and COOT^58,59^. Geometry validation was aided by Molprobity^60^. All ligand restraints were generated with eLBOW^61^ using geometry optimized by quantum mechanical calculations in Gaussian 16 at B3LYP/6-31 g(d,p) level of theory^62^. Final data collection and refinement statistics can be found in Table 2.

### Molecular dynamics (MD) simulations

MD simulations were conducted to investigate the structural and functional dynamics of MPro^WT^ and MPro^D48Y/ΔP168^. The starting structure was derived from the room-temperature X-ray crystal structure of SARS-CoV-2 MPro^WT^ (PDB ID: 6WQF)^11^, and the symmetry operations in PyMOL were used to generate the dimer. The catalytic activity of MPro is critically influenced by the protonation states of the catalytic dyad (H41-C145) and its surrounding residues. Hence, the protonation states of histidine and cysteine residues were assigned based on our previously published neutron diffraction structure (PDB ID: 7JUN^12^). Specifically, H41, H64, H80, and H164 were modeled as charged and doubly protonated at Nε and N_δ_; H163 was protonated at N_δ_, while H172 and H246 were protonated at Nε and were modeled as neutral. Cysteine residues C22, C38, C44, C128, and C145 were modeled as deprotonated thiolates and negatively charged. The dimeric protein system was solvated in a cubic simulation box with periodic boundary conditions, maintaining a minimum distance of 1.2 nm between the protein and the box edges. The net charge of the system was neutralized by adding Na^+^ and Cl^−^ ions to achieve a physiological ionic strength of 150 mM. The TIP3P water model was employed, and all parameters were described using the CHARMM36 force field. Energy minimization was performed to relax the system, followed by equilibration in the NVT ensemble at 300 K for 100 ps and the NPT ensemble at 1 bar for an additional 100 ps. Short-range electrostatic and van der Waals interactions were calculated using a cutoff of 12 Å, while long-range electrostatic interactions were treated with the particle mesh Ewald (PME) method^63,64^. The SETTLE algorithm constrained bond lengths and angles in water molecules, while the LINCS algorithm was used to constrain bonds to hydrogen^65–67^. Production simulations were conducted under isothermal-isobaric conditions for 1 μs using the Parrinello-Rahman barostat to regulate pressure (Table S1)^68^. A time step of 2 fs was employed, and trajectory frames were saved every 200 ps for subsequent analysis. Two independent 1 μs simulations were performed for each protein, with each simulation repeated using a different random seed to introduce slight variations in the initial velocities, thereby enhancing the assessment of reproducibility (Fig. S6). All simulations were performed using GROMACS 2024.4^69^.

### Statistics and reproducibility

Expressed proteins were verified both by DNA sequencing and mass spectrometry. Reproducibility was tested at least 2 times with freshly prepared enzyme and stock solutions of the substrate and inhibitor. Once this was determined to provide consistent reaction rates within an error limit of 5%, the final experiment for the data displayed in the manuscript was carried out in duplicate, and 4 reads per well for each time point. The mean of the data points was used for fitting. The same stock solutions of enzyme and inhibitor were used for ITC and DSF analyses to determine the inhibitor binding constant (*K*_d_)/thermodynamic parameters and thermal denaturation, respectively. Each ITC experiment was carried out with a minimum of 20 injections. DSF experiments were repeated twice in duplicates. *K*_dimer_ by SV-AUC was determined with varying protein concentrations and Lamm equation modeling of the absorbance data. X-ray diffraction data and refinement statistics are shown. Gel images are the best representative for each of the constructs analyzed.

### Reporting summary

Further information on research design is available in the Nature Portfolio Reporting Summary linked to this article.

## Supplementary information

Supplementary Information

Description of Additional Supplementary Files

Supplementary Data 1

Supplementary Data 2

Reporting summary

## Data Availability

The structure and corresponding structure factors have been deposited into the protein data bank with the PDB accession codes 9N6J for MPro^D48Y/ΔP168^, 9N6L for MPro^D48Y/ΔP168^-GC373, 9N6M for MPro^D48Y/ΔP168^-NMV, 9N6N for MPro^D48Y/ΔP168^-PMV, 9N6P for MPro^D48Y/ΔP168^-ESV and 9N6R for MPro^WT^-ESV. Source data files are provided in Supplementary Data 1, Data 2, and Supplementary Information. All other data that support this study are available from the corresponding authors upon reasonable request.
