# Supplementary material for: Characterization of an unusual SARS-CoV-2 main protease natural variant exhibiting resistance to nirmatrelvir and ensitrelvir
Source: Commun Biol. 2025 Jul 17;8:1061. doi: 10.1038/s42003-025-08487-w (PMC12271536; doi:10.1038/s42003-025-08487-w)
Supplement: Supplementary file 1 — Supplementary Information [file 42003_2025_8487_MOESM1_ESM.pdf]

Supplementary Information

Characterization of an unusual SARS-CoV-2 main protease natural variant exhibiting resistance to nirmatrelvir and ensitrelvir

Dipendra Bhandari,[1] Oksana Gerlits,[2] Stephen Keable,[1] Leighton Coates,[3] Annie Aniana,[4] Rodolfo Ghirlando,[5] Nashaat T. Nashed,[4] Andrey Kovalevsky[1]* and John M. Louis[4]*

[1]*Neutron Scattering Division, Oak Ridge National Laboratory, 1 Bethel Valley Road, Oak Ridge, TN, 37831, USA*

[2]*Department of Natural Sciences, Tennessee Wesleyan University, Athens, TN 37303, USA*

[3]*Second Target Station, Oak Ridge National Laboratory, 1 Bethel Valley Road, Oak Ridge, TN, 37831, USA*

[4]*Laboratory of Chemical Physics, National Institute of Diabetes and Digestive and Kidney Diseases, National Institutes of Health, DHHS, Bethesda, MD 20892-0520, USA*

[5]*Laboratory of Molecular Biology, National Institute of Diabetes and Digestive and Kidney Diseases, National Institutes of Health, DHHS, Bethesda, MD 20892-0520, USA*

* Corresponding authors:
Andrey Kovalevsky: kovalevskyay@ornl.gov, John M. Louis: johnl@niddk.nih.gov

Contents

Table S1……………………………………………………………………………….....3
Figure S1…………………………………………………………………………………4
Figure S2…………………………………………………………………………………5
Figure S3…………………………………………………………………………………6
Figure S4…………………………………………………………………………………7
Figure S5…………………………………………………………………………………8
Figure S6…………………………………………………………………………………9
Figure S7………………………………………………………………………………..10
Figure S8………………………………………………………………………………..11
Figure S9………………………………………………………………………………..12
Figure S10..……………………………………………………………………………..13
Figure S11..……………………………………………………………………………..14
References………………………………………………………………………………15

Table S1. **Specific setup details of the MD simulations**

MPro^{WT} (Homodimer of SARS-CoV-2-MPro, PDB: 6WQF)

Number of residues in each protomer	306
Number of solvent molecules (water)	86748
Number of sodium (Na) ions	10
Dimensions of Cubic Simulation Box	14 nm
NaCl Concentration	150 mM (NaCl: 234 Molecules)
Timestep	2 fs
Temperature	300 K
Pressure	1 bar
NVT Equilibration	100 ps
NPT Equilibration	100 ps
MD Simulation Length	1 µs
Water Model	TIP3P
Force Field	CHARMM36
Simulation Package	GROMACS2024.4

MPro$^{D48Y/\Delta P168}$ (Homodimer of SARS-CoV-2-MPro $^{D48Y/\Delta P168}$, PDB: 9N6J)

Number of residues in each protomer	305
Number of solvent molecules (water)	86749
Number of sodium (Na) ions	8
Dimensions of Cubic Simulation Box	14 nm
NaCl Concentration	150 mM (NaCl: 234 Molecules)
Timestep	2 fs
Temperature	300 K
Pressure	1 bar
NVT Equilibration	100 ps
NPT Equilibration	100 ps
MD Simulation Length	1 µs
Water Model	TIP3P
Force Field	CHARMM36
Simulation Package	2024.4

Figure S1.

Amino acid sequence of MPro constructs.

MPro$^{D48Y/\Delta P168}$-GP-6H

```
        10         20         30         40       Y 50         60
SGFRKMAFPS GKVEGCMVQV TCGTTTLNGL WLDDVVYCPR HVICTSEDML NPNYEDLLIR
        70         80         90        100        110        120
KSNHNFLVQA GNVQLRVIGH SMQNCVLKLK VDTANPKTPK YKFVRIQPGQ TFSVLACYNG
       130        140        150        160       P170        180
SPSGVYQCAM RPNFTIKGSF LNGSCGSVGF NIDYDCVSFC YMHHMELPTG VHAGTDLEGN
       190        200        210        220        230        240
FYGPFVDRQT AQAAGTDTTI TVNVLAWLYA AVINGDRWFL NRFTTTLNDF NLVAMKYNYE
       250        260        270        280        290        300
PLTQDHVDIL GPLSAQTGIA VLDMCASLKE LLQNGMNGRT ILGSALLEDE FTPFDVVRQC
    306
SGVTFQGPHH HHHH
```

Calculated molecular weight: 33747.8
Ext. coefficient: 32430 (280 nm)

Precursor $^{(-102)}$MPro$^{D48Y/\Delta P168}$-GP-6H

```
-102              -85        -75        -65        -55        -45
SGKRRVVFNG VSFSTFEEAA LCTFLLNKEM YLKLRSDVLL PLTQYNRDLA LYNKYKYFSG
       -35        -25        -15         -5     1             16
AMDTTSYREA ACCHLAKALN DFSNSGSDVL YQPPQTSITS AVLQSGFRKM AFPSGKVEGC
        26         36         46 Y       56         66         76
MVQVTCGTTT LNGLWLDDVV YCPRHVICTS EDMLNPNYED LLIRKSNHNF LVQAGNVQLR
        86         96        106        116        126        136
VIGHSMQNCV LKLKVDTANP KTPKYKFVRI QPGQTFSVLA CYNGSPSGVY QCAMRPNFTI
       146        156        166 P      176        186        196
KGSFLNGSCG SVGFNIDYDC VSFCYMHHME LPTGVHAGTD LEGNFYGPFV DRQTAQAAGT
       206        216        226        236        246        256
DTTITVNVLA WLYAAVINGD RWFLNRFTTT LNDFNLVAMK YNYEPLTQDH VDILGPLSAQ
       266        276        286        296        306
TGIAVLDMCA SLKELLQNGM NGRTILGSAL LEDEFTPFDV VRQCSGVTFQ GPHHHHHH
```

Calculated molecular weight: 46463.2
Ext. coefficient: 42670 (280 nm)

Figure S1. **Amino acid sequence of recombinant MPro constructs used in this study and their designations.** Non-native residues at the C-terminus are underlined. Mutations are indicated in red letters above the wild-type sequence. Theoretical mass and extinction coefficient of the proteins are indicated below the sequence of the corresponding construct. $^{(-102)}$ denotes 102 amino acids of the C-terminal residues of nsp4, flanking the N-terminus of nsp5 (MPro), appended to MPro. The precursor construct undergoes N-terminal autoprocessing upon expression to release MPro$^{D48Y/\Delta P168}$-GP-6H. The 6H-tag at the C-terminus of MPro enables isolation by nickel-affinity chromatography followed by HRV protease cleavage to remove the 6H-tag in both constructs. Based on the simple rule that when the residue following Met is a Gly or Ser, in all constructs, the N-terminal Met is fully excised by methionyl-aminopeptidase as verified by mass spectrometry [1, 2].

Figure S2.

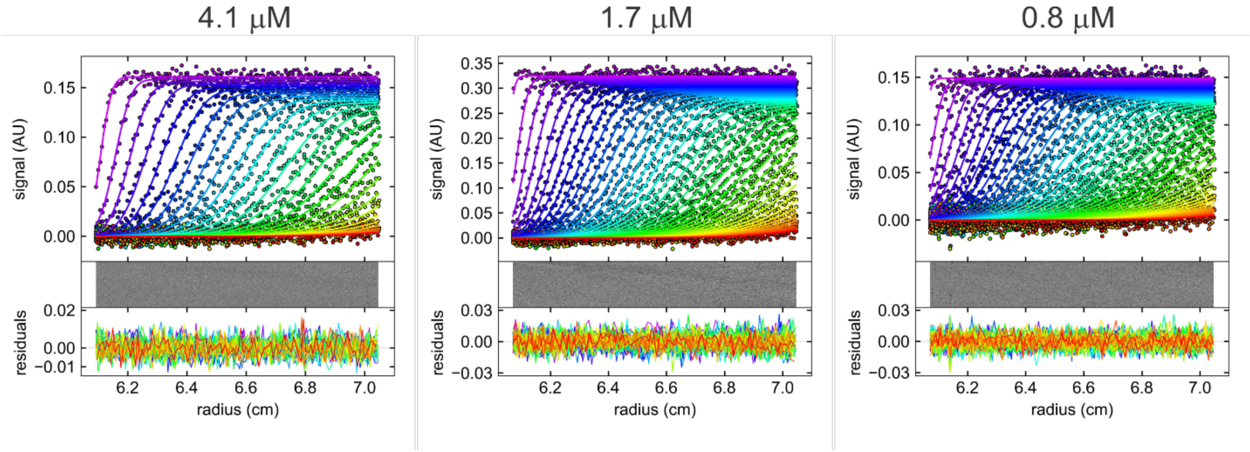

Figure S2. **Lamm equation modeling to determine the K_{dimer} of MPro$^{D48Y/\Delta P168}$.** Absorbance sedimentation velocity data collected for MPro$^{D48Y/\Delta P168}$ at 50,000 rpm and 25°C in 12 mm pathlength cells, as noted in Figure 2, were analyzed globally in terms of Lamm equations describing a reversible monomer-dimer self-association. Data for 4.1 μM (280 nm), 1.7 μM (230 nm), and 0.8 μM (230 nm) of MPro$^{D48Y/\Delta P168}$ are shown from left to right. For clarity, only every third scan and every third data point are shown. Solid lines through the experimental points show the best fit to the monomer-dimer model. A bitmap representation of the residuals to the best fit is shown below each plot, together with the overlaid residual plots. The analysis returns a monomer-dimer dissociation constant of 0.06 ± 0.01 μM, with the error representing a 68% confidence interval.

Figure S3.

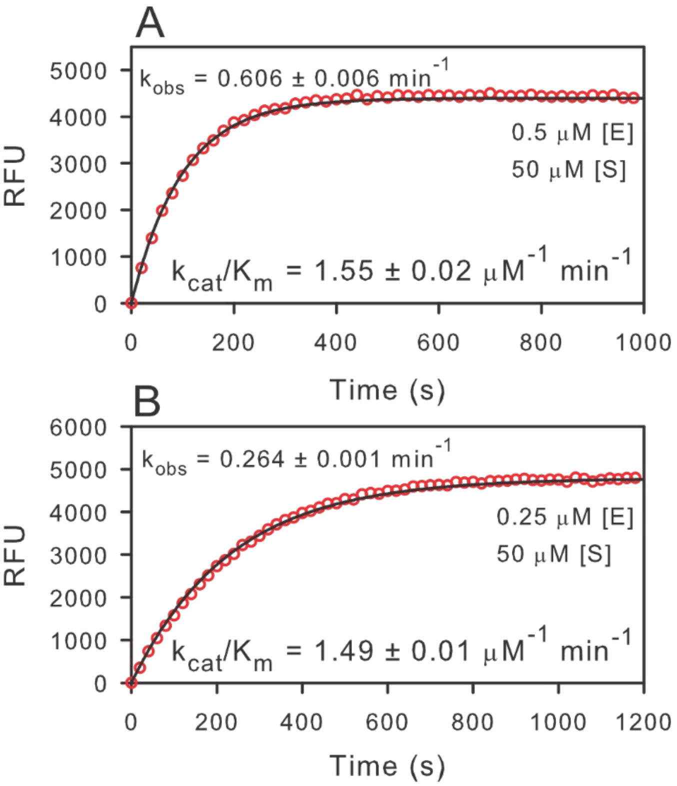

Figure S3. **Catalytic activity of MPro$^{D48Y/\Delta P168}$.** Enzyme assays were carried out in duplicate as described in Methods. Since the catalytically active form of the enzyme is dimeric [3], the observed first-order rate constant ($k_{obs} = V_{max}/K_m$) is $k_{cat}[E_o\text{-}E]/K_m$, where E_o and E denote the total protein and monomer concentration, respectively. Correlation coefficients (R^2) were observed in the range of 0.9947-0.9986. Dimer populations at 0.5 (**A**) and 0.25 (**B**) μM were calculated based on the mass action law using a K_{dimer} of 0.06 ± 0.01 μM derived from SV-AUC experiments (see Fig. 2 and S2). The calculated dimer form of the protein at 0.5 and 0.25 μM is 0.392 and 0.178 μM, respectively [4].

Figure S4.

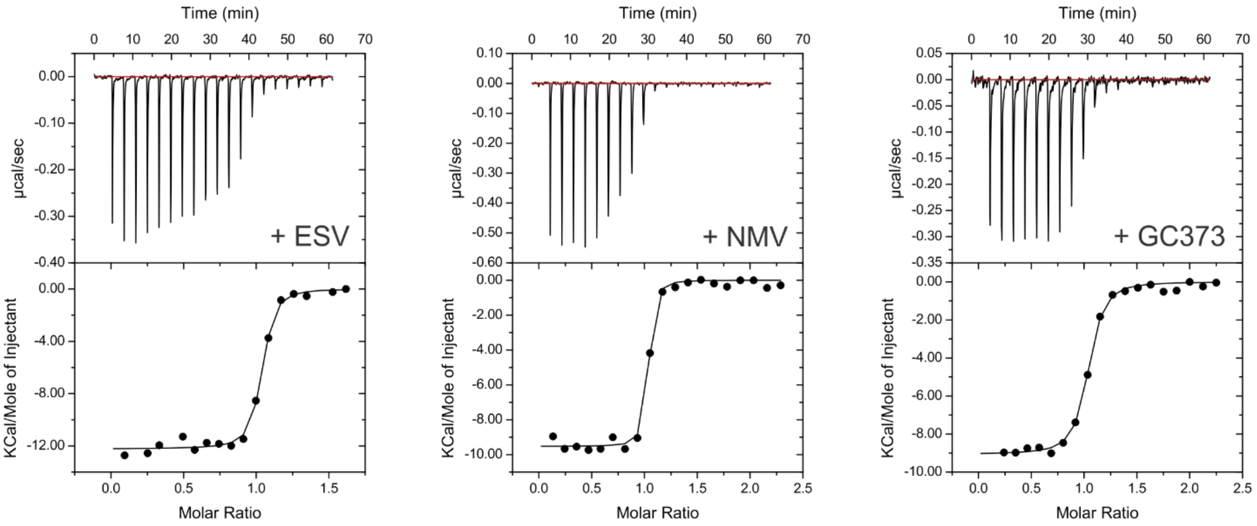

Figure S4. **Binding isotherms of inhibitors to MPro$^{D48Y/\Delta P168}$.** Titrations were carried out in buffer B (25 mM Tris-HCl, pH 7.2, 20 mM NaCl and 1 mM TCEP) at 28 °C with 30 µM protein in the cell and inhibitor (in the syringe) at 10 times the protein concentration. Data were processed using the Origin software provided with the instrument. The inhibitor dissociation constant (K_d) and thermodynamic parameters are listed in Table 1.

Figure S5.

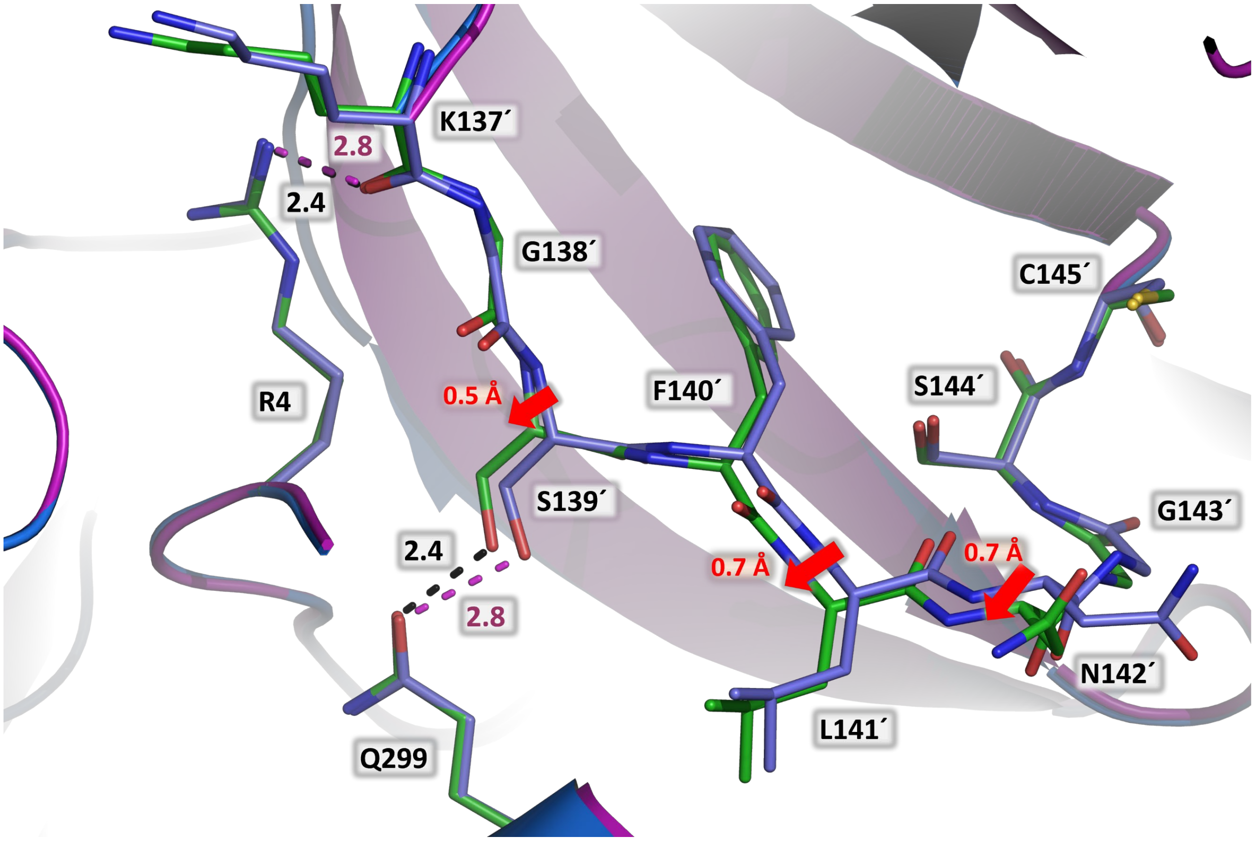

Figure S5. **Superposition of MPro^{WT} and MPro$^{D48Y/\Delta P168}$.** The dimer interface made by R4 and Q299 with the residues of the oxyanion loop of the other protomer is shown. In MPro$^{D48Y/\Delta P168}$, the hydrogen bonds between the protomers are shorter, thus stronger. MPro$^{D48Y/\Delta P168}$ and MPro^{WT} are depicted with carbon atoms colored green and purple, respectively. Distances are in Angstrom.

Figure S6.

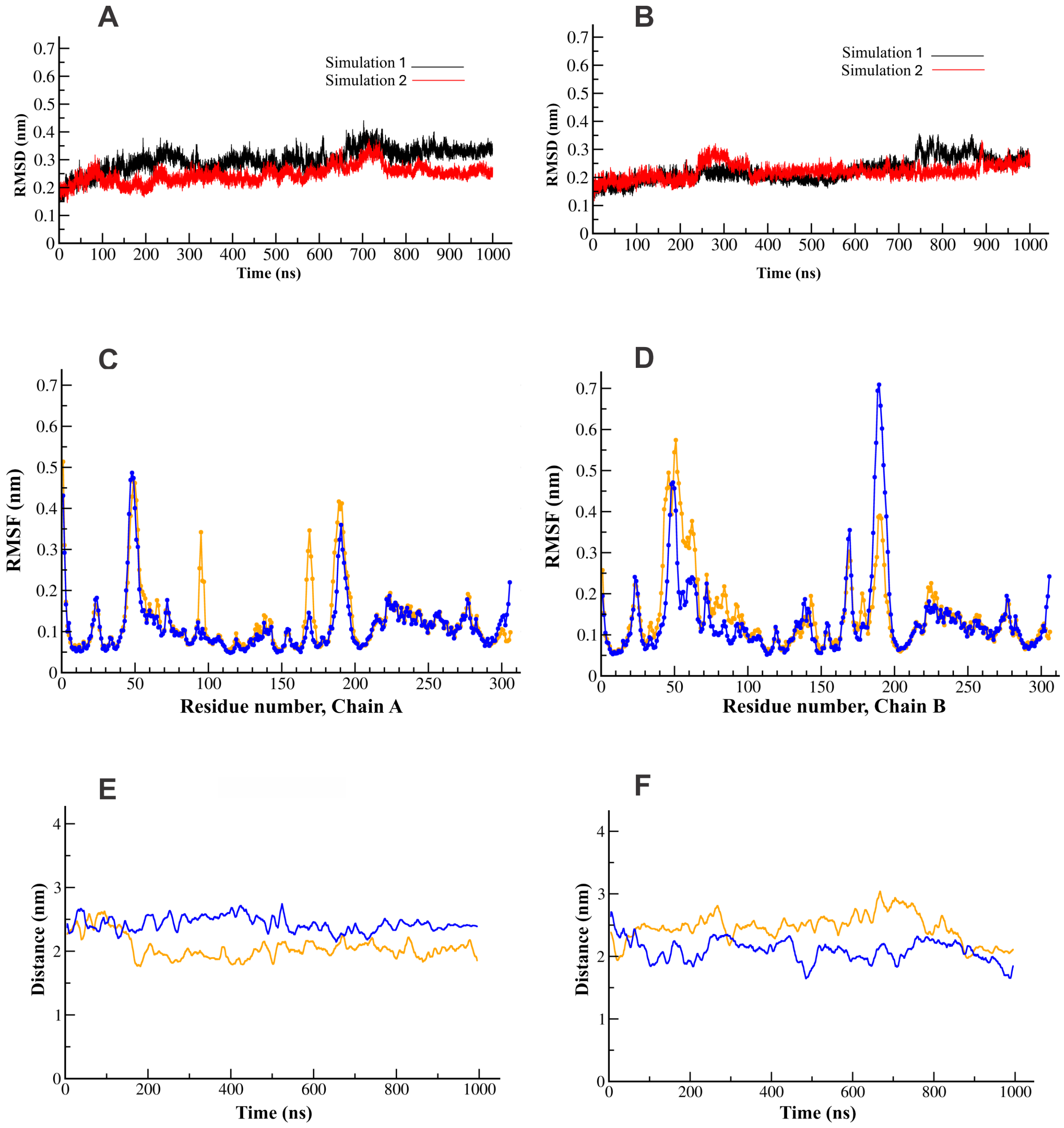

Figure S6. **Comparison of structural dynamics between the MPro^{WT} and MPro$^{D48Y/\Delta P168}$.** Root-mean-square deviation (RMSD) profiles of backbone atoms across all simulation trajectories for MPro^{WT} (**A**) and MPro$^{D48Y/\Delta P168}$ (**B**). Root-mean-square fluctuation (RMSF) profiles highlighting residue-specific flexibility for protomer A (**C**) and protomer B (**D**). Alpha-carbon distances between residues 47 of the short subsite S2 helix and residue 169 at the tip of the subsite S4 β-hairpin loop in protomer A (**E**) and protomer B (**F**). The distances are presented as running averages of 1,000 frames (2fs/frame) obtained from the MD simulations, highlighting structural variations. The profiles and distances in panels **C-F** are averaged over two 1μs-long simulations for MPro^{WT} (orange) and MPro$^{D48Y/\Delta P168}$ (blue).

Figure S7.

Covalent Inhibitors:

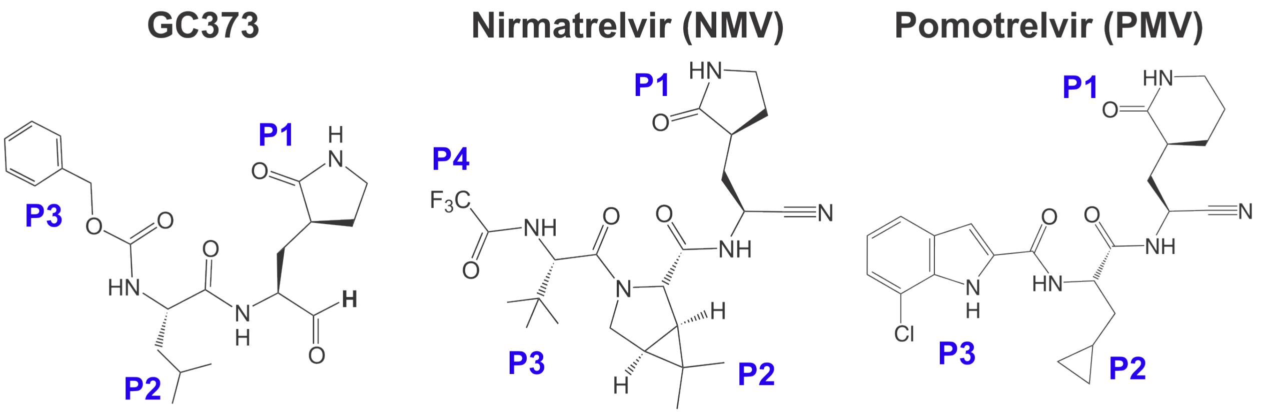

Noncovalent Inhibitor:

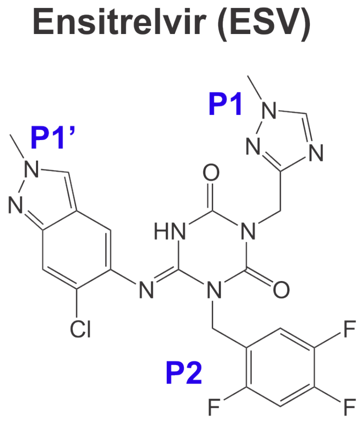

Figure S7. **Chemical diagrams of the inhibitors used in this study.** P4 to P1′ denote positions in the substrate sequence corresponding to substrate binding subsites S4 through S1′.

Figure S8.

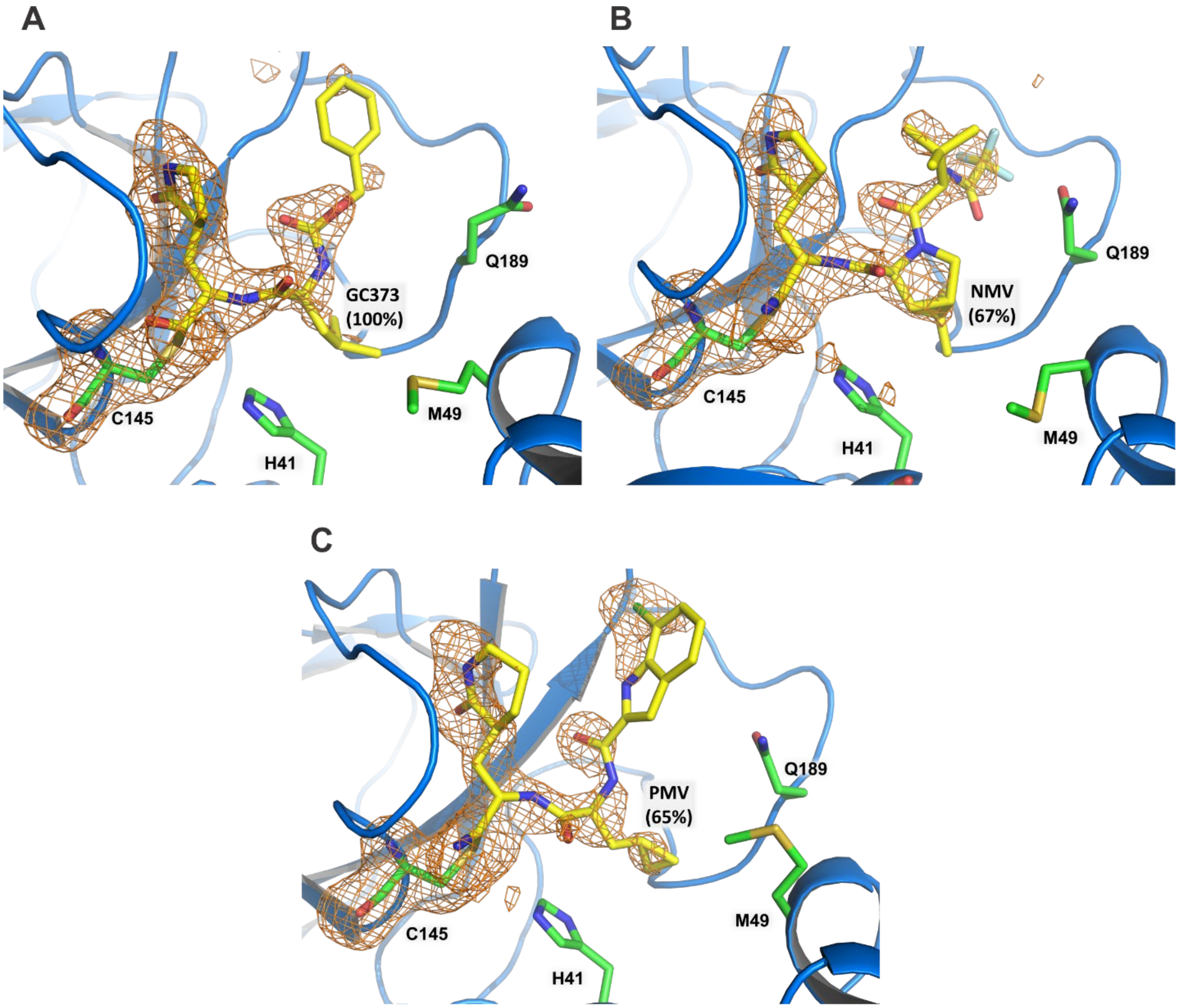

Figure S8. **Room-temperature X-ray structures of MPro$^{D48Y/\Delta P168}$ in complex with inhibitors GC373 (A), NMV (B), and PMV (C).** Electron density is shown as Polder omit maps (orange mesh contoured at 2.5 σ), with the refined occupancy of each ligand shown as a percentage.

Figure S9.

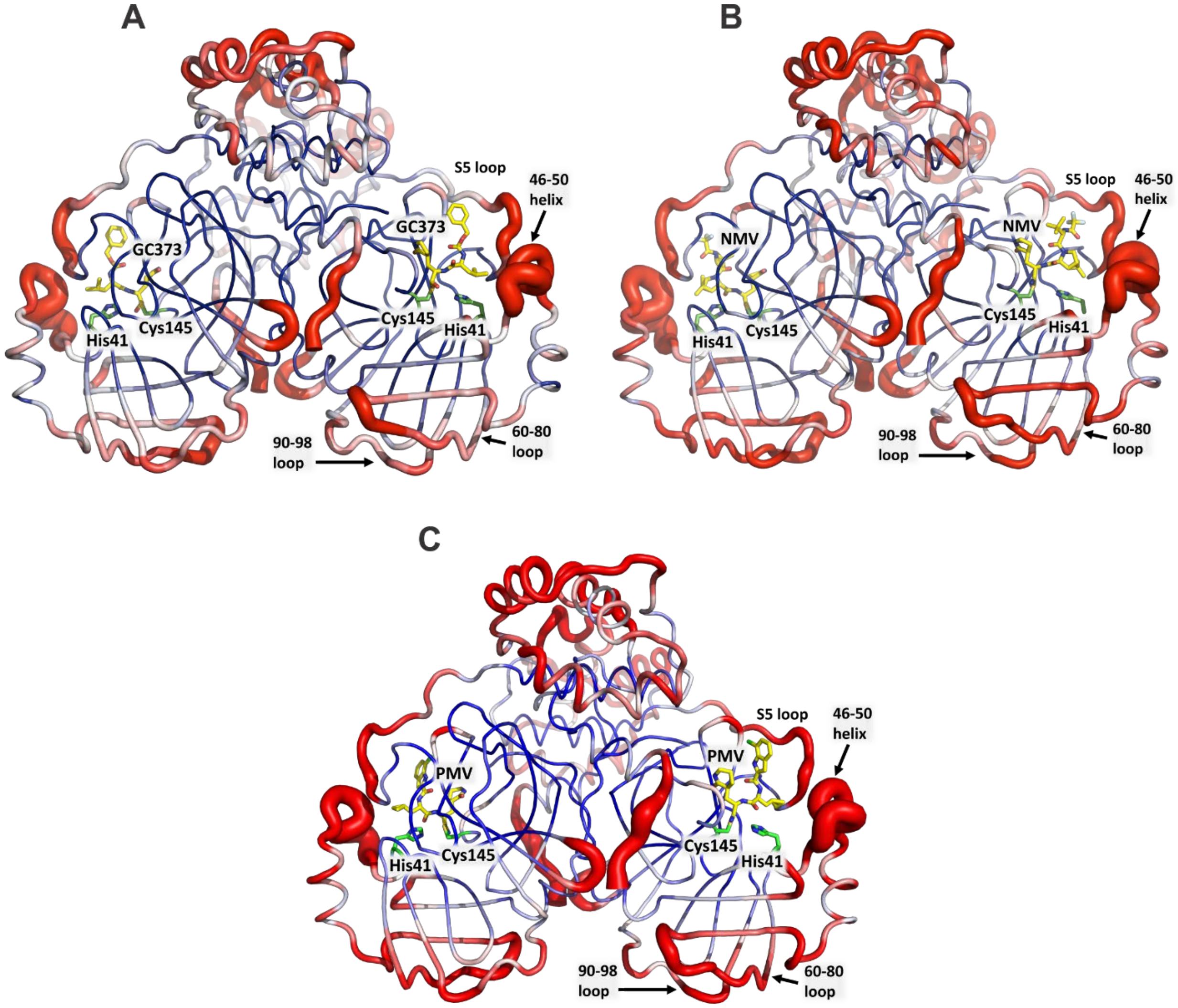

Figure S9. **Dynamics of inhibitor-bound MPro$^{D48Y/\Delta P168}$ in complex with GC373 (A), NMV (B), and PMV (C) based on the B-factor analyses.** The structures are shown in cartoon-putty representations colored by backbone atomic displacement parameters (B-factors). Redder and thicker putty depicts higher B-factors.

Figure S10.

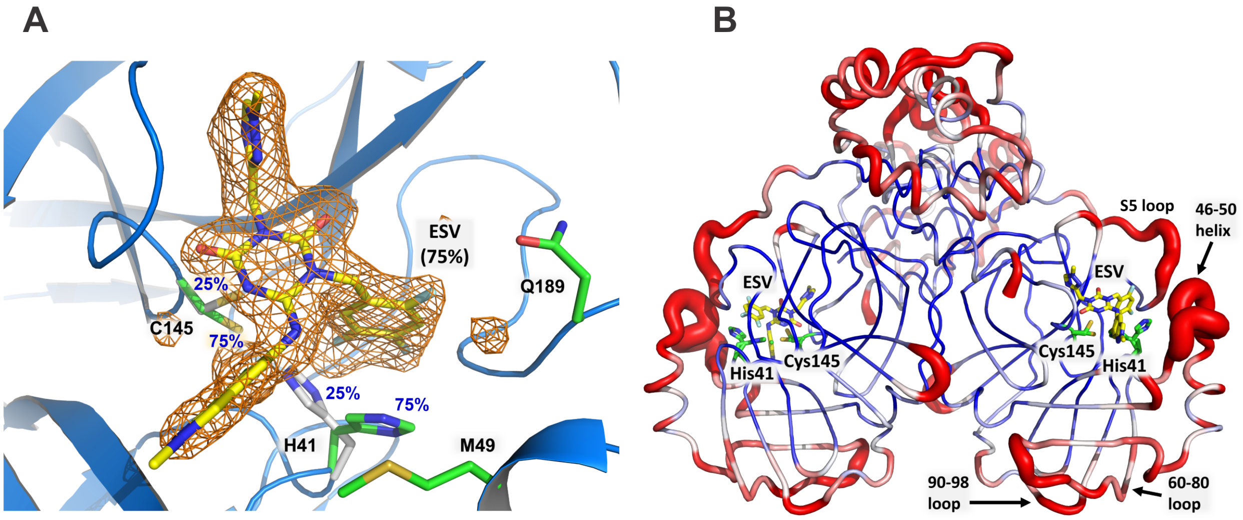

Figure S10. **Room-temperature structure of MPro$^{D48Y/\Delta P168}$-ESV complex**. (**A**) Electron density of ESV is shown as a Polder omit map (orange mesh contoured at 3.0 σ) with the refined occupancy of ESV shown as a percentage. (**B**) Cartoon-putty representation is colored by backbone atomic displacement parameters (B-factors). Redder and thicker putty depicts higher B-factors.

Figure S11.

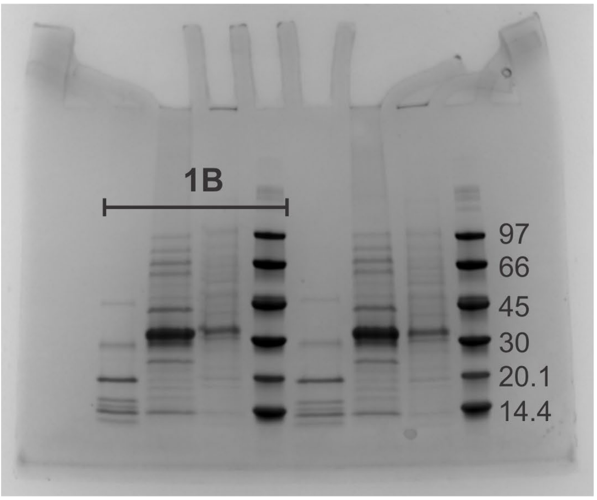

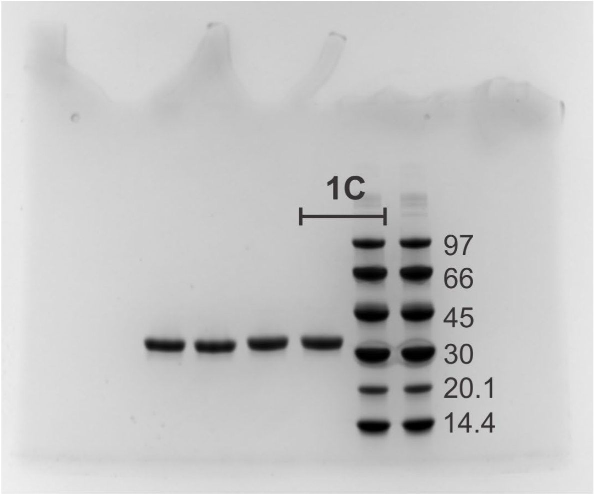

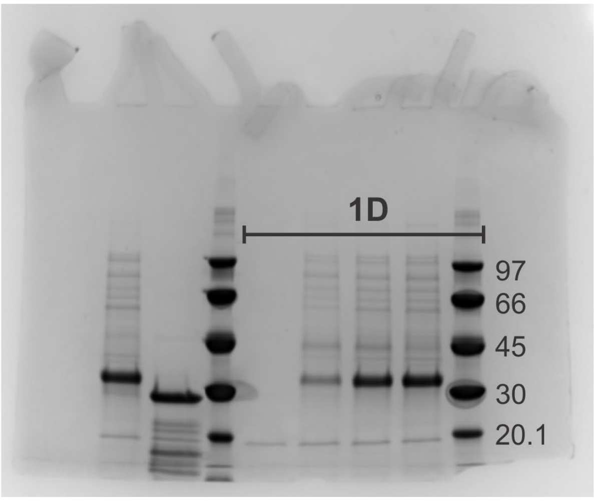

Figure S11. Uncropped gel images of Fig. 1B, 1C and 1D.

References

1. Wingfield PT. N-Terminal Methionine Processing. *Curr Protoc Protein Sci* **88**, 6 14 11-16 14 13 (2017).

2. Hirel PH, Schmitter MJ, Dessen P, Fayat G, Blanquet S. Extent of N-terminal methionine excision from Escherichia coli proteins is governed by the side-chain length of the penultimate amino acid. *Proc Natl Acad Sci U S A* **86**, 8247-8251 (1989).

3. Nashed NT, Aniana A, Ghirlando R, Chiliveri SC, Louis JM. Modulation of the monomer-dimer equilibrium and catalytic activity of SARS-CoV-2 main protease by a transition-state analog inhibitor. *Commun Biol* **5**, 160 (2022).

4. Schuck P. On the analysis of protein self-association by sedimentation velocity analytical ultracentrifugation. *Anal Biochem* **320**, 104-124 (2003).
